# Bridging Plant and Human Radiation Response and DNA Repair through an In Silico Approach

**DOI:** 10.3390/cancers9060065

**Published:** 2017-06-06

**Authors:** Zacharenia Nikitaki, Athanasia Pavlopoulou, Marcela Holá, Mattia Donà, Ioannis Michalopoulos, Alma Balestrazzi, Karel J. Angelis, Alexandros G. Georgakilas

**Affiliations:** 1 DNA Damage Laboratory, Physics Department, School of Applied Mathematical and Physical Sciences, National Technical University of Athens, Iroon Polytechniou 9, 15780 Zografou, Greece; znikitaki@mail.ntua.gr; 2 Department of Computer Science and Biomedical Informatics, University of Thessaly, Papasiopoulou 2-4, 35100 Lamia, Greece; athanasiapavlo@gmail.com; 3 Institute of Experimental Botany ASCR, Na Karlovce 1, 16000 Praha, Czech Republic; hola@ueb.cas.cz (M.H.); angelis@ueb.cas.cz (K.J.A.); 4 Gregor Mendel Institute (GMI) Austrian Academy of Science, Vienna Biocenter (VBC), Dr. Bohr Gasse 3, 1030 Vienna, Austria; mattia.dona@gmi.oeaw.ac.at; 5 Centre of Systems Biology, Biomedical Research Foundation, Academy of Athens, 11527 Athens, Greece; imichalop@bioacademy.gr; 6 Department of Biology and Biotechnology ‘Lazzaro Spallanzani’, via Ferrata 1, 27100 Pavia, Italy; angelis@ueb.cas.cz

**Keywords:** DNA damage repair, in silico analysis, bioinformatics, ionizing radiation, ultraviolet radiation, plant radiation biodosimeter

## Abstract

The mechanisms of response to radiation exposure are conserved in plants and animals. The DNA damage response (DDR) pathways are the predominant molecular pathways activated upon exposure to radiation, both in plants and animals. The conserved features of DDR in plants and animals might facilitate interdisciplinary studies that cross traditional boundaries between animal and plant biology in order to expand the collection of biomarkers currently used for radiation exposure monitoring (REM) in environmental and biomedical settings. Genes implicated in trans-kingdom conserved DDR networks often triggered by ionizing radiation (IR) and UV light are deposited into biological databases. In this study, we have applied an innovative approach utilizing data pertinent to plant and human genes from publicly available databases towards the design of a ‘plant radiation biodosimeter’, that is, a plant and DDR gene-based platform that could serve as a REM reliable biomarker for assessing environmental radiation exposure and associated risk. From our analysis, in addition to REM biomarkers, a significant number of genes, both in human and *Arabidopsis thaliana,* not yet characterized as DDR, are suggested as possible DNA repair players. Last but not least, we provide an example on the applicability of an *Arabidopsis thaliana—*based plant system monitoring the role of cancer-related DNA repair genes *BRCA1*, *BARD1* and *PARP1* in processing DNA lesions.

## 1. Introduction

Both prokaryotic and eukaryotic cells exposed to radiation acquire different types of DNA lesions (e.g., single-strand breaks (SSB), double-strand breaks (DSB), mismatches, modified bases etc.). This genotoxic effect induced by radiation leads to the activation of DNA damage response (DDR) pathways. DDR can be defined as the sum of functions (sensors, transducers, effectors) that orchestrate DNA damage sensing and signal transduction, triggering either DNA repair, cell survival or cell death. DDR represents a prominent example of ‘DNA self-awareness’ or ‘chemical intelligence’ [1], since this evolutionarily conserved mechanism maintains genome integrity by releasing qualitative/quantitative information on a wide range of DNA lesions and activate proper responses (lesion-specific DNA repair enzymes/pathways) [1]. Of particular importance, DDR deregulation is linked to common human diseases such as cancer [2,3].

During evolution, plants have acquired a range of genes encoding products that either participate in one or more DNA repair pathways with distinct spatial/temporal expression profiles, or contribute to complex signaling pathways that mediate DNA damage sensing and activation of DDR. The conserved features of DDR might facilitate interdisciplinary studies that cross the traditional boundaries between animal and plant biology, with the aim of expanding the collection of DNA damage biomarkers currently used in environmental and biomedical settings. We suggest the potential application of a ‘plant radiation biodosimeter’ as a potential tool for exploiting plant DDR genes selected as radiation exposure monitoring (REM) biomarkers for assessing radiation risk in environments. In all cases of radiation exposure or even radiomimetic drugs, complex DNA damage is expected to be induced triggering various DNA repair pathways [4]. The main pathways for DSB repair are homologous recombination (HR) and the less precise non-homologous end joining (NHEJ). Recent advances in the discovery of new repair factors suggest differences between the human and plant systems and more insights needed. For example, a new element of the NHEJ, human PAXX (PAralog of XRCC4 and XLF, also called C9orf142) has been recently identified as a new XRCC4 superfamily member with its crystal structure to resemble that of XRCC4. PAXX has been shown to interact directly with the DSB-repair protein Ku and to be recruited at DNA-damage sites in cells [5]. As recently discussed in [6], in most cases of genotoxic stress where DSBs occur, emphasis is given on the repair of DSBs and especially at the chromatic level. Recent discoveries of new factors (like PAXX) which operate at the level of chromatin, emphasizes the concept of a dominant role of chromatin structure in the regulation of cellular DDR regulation [6].

In addition, other features of an efficient biological system/platform used as a radiation biodosimeter would be to be able to follow DNA damage related chromatin changes like nucleosome remodeling, variant histone exchange, non-histone chromatin protein mobility alteration and histone tail post-translational modification as reviewed in [7].

Herein, we describe the design of an *in silico* method to identify and rescue the best candidate genes for the plant radiation biodosimeter by conducting a comparative analysis of genes implicated in DDR both in animals and plants. Towards this end, the DDR-related genes of human and *Arabidopsis thaliana*, representing the animal and plant kingdom, respectively, were compared.

## 2. Plant Radiation Biodosimeter

The application of radiation exposure biomarkers is essential for evaluating cytotoxicity and genotoxicity in the human tissues in radiation oncology, as well as for biodosimetry purposes in the case of nuclear catastrophe and accidental radiation exposure. Recent extensive comparative studies on different biodosimetry approaches on the same irradiated cells underlines the necessity of employing a multiparametric approach to accomplish an accurate dose and risk estimation [8].

Plants, unlike mammals, exhibit an inherent radioresistance, a feature considered as a limiting factor for their application as ‘radiation biodosimeters’. There are many similarities between plants and humans in response to radiation. The ATR and ATM protein kinases are recognized as key players in a variety of responses to DNA damaging agents [9]. The *Arabidopsis thaliana* genome includes both ATR and ATM orthologs, and plants with null alleles of these genes are viable. *Arabidopsis* ATR/ATM mutants display hypersensitivity to γ-irradiation similarly to humans [10]. Radiation sensitivity is often associated with reduced ability to efficiently repair DSBs and/or activate DDR as reported by Borràs-Fresneda et al. [11] who compared for example radiation response in radiosensitive and radioresistant lymphocytes.

The molecular bases of natural radiotolerance have been investigated in the IR-resistant fungus *Ustilago maydis* which relies on the presence of a highly efficient machinery homologous recombinational (HR) repair of DNA damage, and particularly on the activity of the *BRH2* gene, homolog of the human *BRCA2* gene [12]. Recently, the *γ*-rays responsive transcriptome of the radioresistant basidiomycetous fungus *Cryptococcus neoformans* has been identified by Jung et al. [13] who found a novel transcription factor containing a basic leucine zipper domain, named BDR1 (bZIP TF for DNA damage response), able to modulate the expression of DNA repair genes. The *BDR1* gene expression was in turn regulated by the highly conserved DDR protein kinase RAD53 [13]. Animals and plants display different levels of radiosensitivity, with a radiotolerance range of 0.001–1 and 1–100 Gy, respectively [14]. Plants have been exposed to IR, which is part of the natural background radiation, throughout evolution, with the consequent enhancement of DNA repair mechanisms necessary to cope with genotoxic stress. It has been reported that radioresistance positively correlates with genome size, since polyploidy facilitates protection against DNA damage [14,15]. Coniferous trees (e.g., pine trees) are very radiosensitive and they show severe damage leading to mortality when exposed to doses >17 Gy. On the contrary, deciduous trees (e.g., birch, alder and aspen) shed their irradiated foliage on the ground and thus can withstand radiation doses up to 90 Gy. The most radiotolerant plants are the herbaceous (weeds) and pasture plants (e.g., grasses and legumes) which are able to withstand doses up to 870 Gy [16,17].

### 2.1. Bioinformatics Approaches for the Identification of Candidate Genes for the Plant Radiation Dosimeter

The basic idea was to discover through *in silico* analysis those genes that could serve as reliable biomarkers or a ‘global tool’ for the development of a ‘non-mammalian radiation dosimetry’. Using meta-analysis and bioinformatics procedures, Nikitaki et al. [18,19] have recently identified unique human gene biomarkers specific for different types of cellular stresses (e.g., IR, replication or oxidative stress), pointing out the potential application, in terms of experimental exploitation and technical advancement, of the selected gene products in the detection of harmful environmental stresses. The investigation described herein has been expanded to develop an in silico-plant-based platform, useful for REM in a non-mammalian and relatively inexpensive model, such as plants (‘the plant radiation dosimeter’). From a biophysical point of view, IR and non-IR exhibit substantially different DNA damage patterns, thus inducing different DDR pathways dominated by distinct genes/proteins [20]. The expected differences in radiation-induced damage at the protein/lipid level could also account for different profiles of gene induction. To this end, we focused on those genes encoding products that serve as ‘exclusive’ biomarkers for the in planta detection of radiation exposure and identification of radiation quality. These universal biomarkers could be expressed in different plant populations/communities specifically linked to different geographical areas. As for radiation quality, attention was given to genes responsive to X-rays, *γ*-rays and non-IR (UV-A, UV-B or UV-C). In silico searches were performed on a plethora of plant species based on Gene Ontology (GO) terms. These GO terms served as filters in plant databases and, for each plant species, the corresponding gene lists were connected to the model plant *Arabidopsis thaliana*, based on their orthologous counterpart. Based on a protein-protein interaction network, we detected the most important orthologues in terms of functionality (nodes). The final sorting was performed according to orthologue multiplicity (number of orthologues in different species). The detailed procedure is described below.

### 2.2. Screening Strategy and Final Selection of Candidate Genes

#### 2.2.1. Selection of Gene Ontology Terms

**Figure 1:**
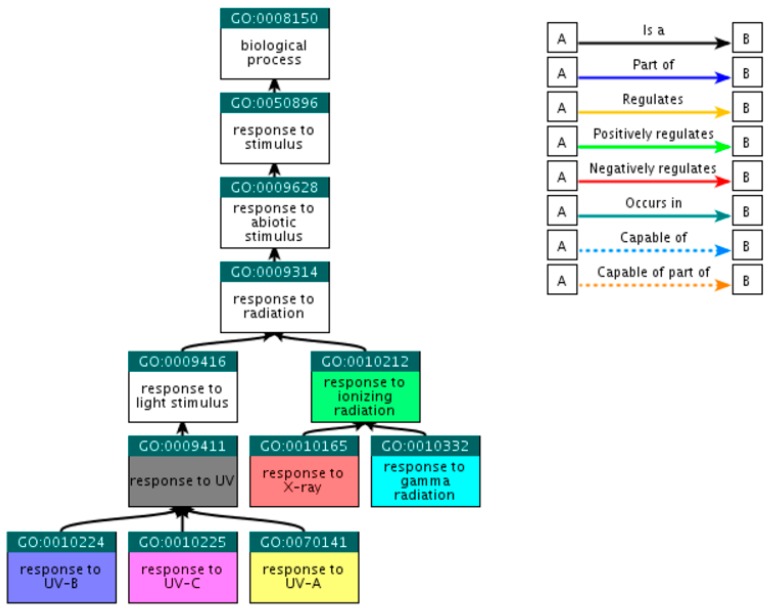
Ancestor tree showing the hierarchical relations of the seven selected GO terms. The 48 rest child terms, referred in the text, are not presented.

Beginning with the broad GO term ‘response to radiation’ through the *QuickGO* platform (http://www.ebi.ac.uk/QuickGO) [21], child GO terms that fall within the selected criteria were explored. Since *QuickGO* provides only the direct descendants of each term, the search for direct descendants for the resultant GO terms was repeated until all child terms of the initial input were collected. At this step, 55 GO terms were retrieved, among which only 7 were eligible as sufficient, i.e., there was no need of including further child terms (Table 1). The hierarchical relations of the selected GO terms are presented at Figure 1, including the initial GO term ‘response to radiation’; however, this term was not included in the final selection.

#### 2.2.2. Orthologous Genes

In order to detect reliable and comprehensive biomarkers of assessing radiation exposure, all the available plant species from the *Ensembl Plants* annotation system [22] were investigated for genes orthologous to the reference plant *A. thaliana*. For each GO term and for every available plant (a total of 39, including the model plant), the counterpart of each orthologue was found in *A. thaliana*. At this step, 273 lists of genes were derived, that is, the product of the 39 plants with the seven selected GO terms. For each GO term, the corresponding 39 lists were unified, ending up with seven sets of genes with a total number of 410 different genes.

#### 2.2.3. Exclusion of Common Genes

**Figure 2:**
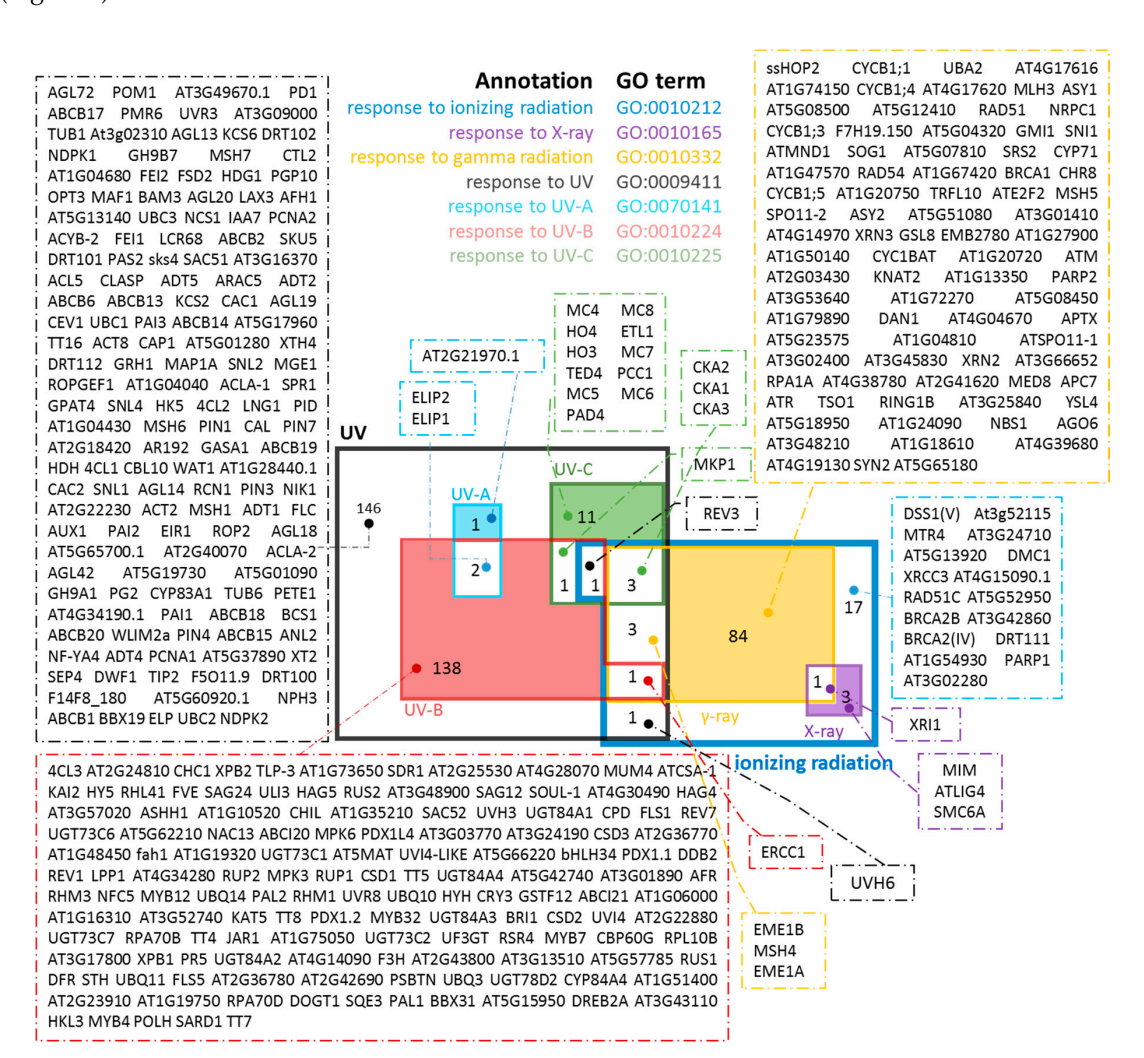
Preliminary screening of suitable genes. Venn diagram of retrieved orthologous genes from all different plant species and for specific GO (Gene Ontology) terms. The colored regions are the suggested gene pools used for further screening, as described in Figure 3.

Given that in this study biodosimeters specific for radiation-quality are sought, genes categorized into more than one of the desired radiation subsets (X-ray, *γ*-ray, UV-A, UV-B and UV-C) had to be excluded from the subsequent steps of the analysis. Using the *Draw Venn* application [23], the corresponding lists of intersections among all the possible combinations of the sets were provided (Figure 2).

#### 2.2.4. Protein-Protein Interactions Network

The initial screening resulted into an overall number of candidate genes that was too large to be informative. In order to reduce this number, STRING v10 (http://string-db.org) [24] was utilized. STRING V.10.0 is a database and a browser that for a given set of genes provides protein-protein interaction networks, allowing the user to set the criteria of the interaction prediction methods.

**Figure 3:**
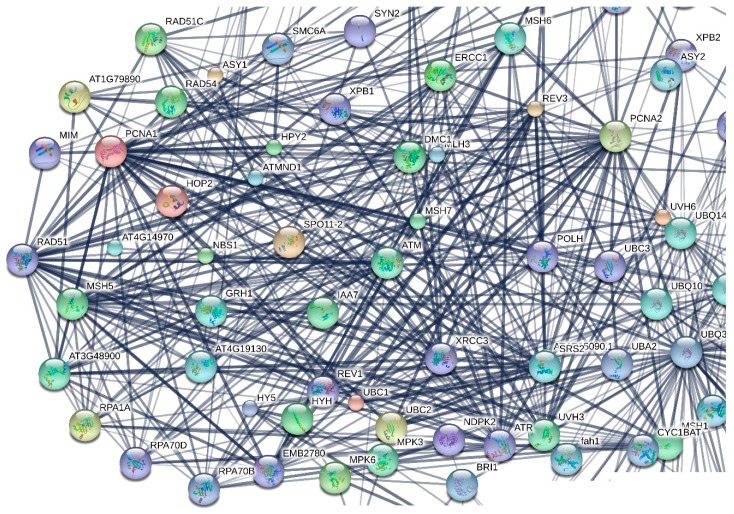
Detail of the protein-protein interaction network, created using STRING V.10.0, where the 237 *A. thaliana* selected by the previous step genes were set as input. The network was rearranged in order to better identify some of the key genes.

The resulting protein interaction network (a detail of which is presented in Figure 3) revealed genes/gene products that serve as nodes of dense cliques and then those genes were selected as the most critical ones for the plant functionality. The selected genes are presented in Table 2.

#### 2.2.5. Ranking Based on Multiplicity

To further reduce the number of suggested genes, candidates highlighted in the previous step were ranked according to their multiplicity of incidence among the plant species under study. Genes that have orthologues in most of the plant species under investigation (Table 2) were used for establishing the plant radiation dosimetry.

#### 2.2.6. Human Orthologues of the Resulting Genes

The candidate genes retrieved through in silico analyses could be also experimentally validated. Towards this end, first, their distinct expression profiles in relation to the different regions of the electromagnetic spectrum need to be estimated in order to determine radiation-specific gene up-regulation. In this way, only those genes induced at a certain wavelength range would be included in the plant radiation dosimeter. Experimental validation would allow to elucidate the function of genes that are annotated to a GO term. For instance, a given gene which is annotated to the GO term ‘response to UV-B’ could likely respond to UV-C as well. Conversely, if a given gene is not annotated to a specific GO term does not necessarily mean that the gene is not classified under this term, but it has rather not been verified yet. Last but not least, in order to provide a better understanding of the ‘plant biodosimeter genes’, we searched for orthologues of the proposed plant genes (Table 2) the products of which could serve as biodosimeters in human (Table 3). In addition, we examined whether the human orthologous genes are annotated to any of the initial GO terms (Table 1), that is, we examined whether the human orthologues shown in Table 3 have been also characterized as genes involved in the response to radiation. To this end, for the genes in the second column of Table 3, the RefSeq [25] accession code of their corresponding encoded proteins was identified (Table 3, third column). For every protein, OrthoGroups were found in OrthoMCL-DB [26] (Table 3, fourth column) and scanned for orthologues in *H. sapiens.* The human proteins, along with their Ensembl protein identifiers (i.e., ENSP) [26], are presented in the fifth column of Table 3. The corresponding gene names (according to HUGO gene nomenclature (HGNC) [27]), were assigned to the retrieved proteins (Table 3, sixth column). Notably, among these proteins, there are also several key DSB repair proteins like RAD54, RAD51, LIG4 (DNA LIGASE 4), as well as proteins participating in HR, MMR (e.g., MSH5 or MutS-HOMOLOG 5), NER (ERCC5, ERCC3; or EXCISION REPAIR CROSS-COMPLEMENTING 5 and 3, respectively) and DDR (RPA1 or REPLICATION PROTEIN A1), further highlighting the pivotal role of the DNA damage repair components in optimized radiation biodosimetry.

## 3. Comparison between *Arabidopsis thaliana* and *Homo sapiens* DNA Repair Mechanisms

Given that DDR pathways are the principle molecular pathways triggered following exposure to IR and non-IR, both in mammals and plants, the DNA repair mechanisms were analyzed comparatively in the model plant and animal species, *Arabidopsis thaliana* and human, respectively.

### 3.1. Comparative Analysis Strategy

#### 3.1.1. Selection of Gene Ontology Terms

**Figure 4:**
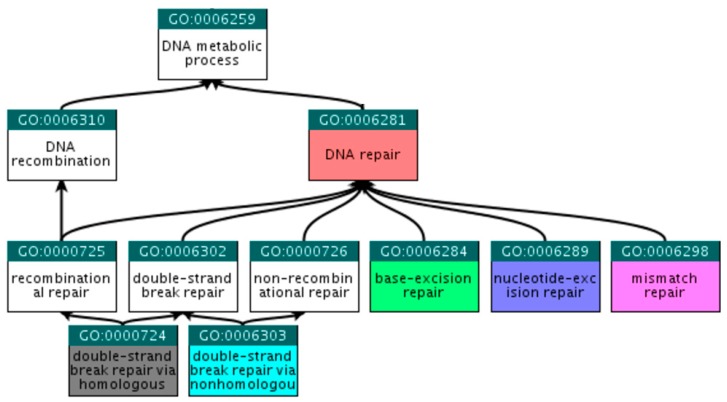
Ancestor tree showing the hierarchical relations of the six selected GO terms.

At the QuickGO (http://www.ebi.ac.uk/QuickGO) [21] platform, child terms that fall within the selected criteria were explored, beginning with the broad term ‘DNA repair’. Following steps similar to those described in Section 2.2.1, we ended up with the six GO terms presented in Table 4 and Figure 4.

#### 3.1.2. Human DNA Repair Genes

**Figure 5:**
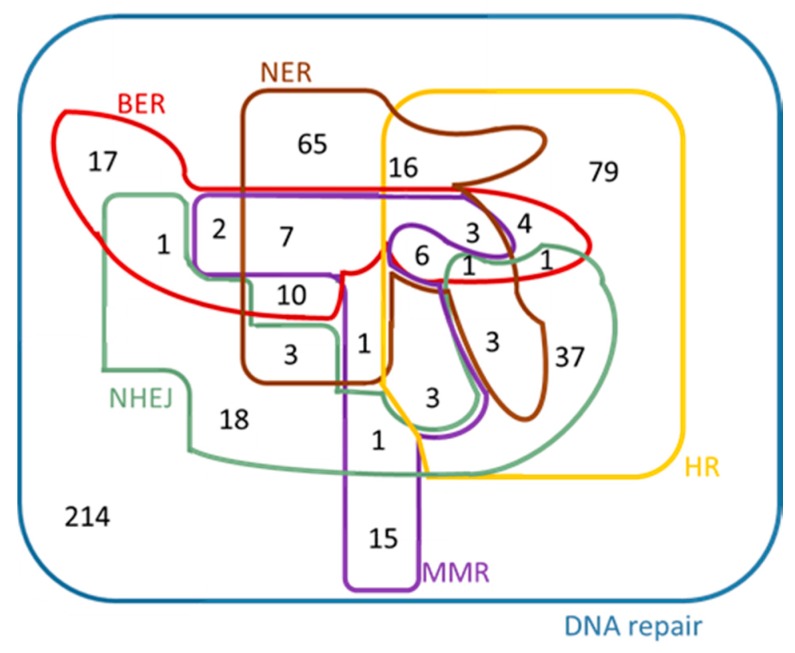
Venn diagram of the *Homo sapiens* genes that were found under each of the GO terms listed in Table 4.

From Ensembl [28], Biomart, Ensembl Genes 83, the dataset ‘*Homo sapiens* genes (GRCh38.p5)’ was chosen. For each search a separate GO term listed in Table 4 was used as ‘Filter’. By choosing ’Ensembl Gene ID’ and ‘HGNC symbol’ under ‘Attributes – Features’, six ‘.txt’ files were created for *Homo sapiens.* The Venn diagram of the initial genes is presented in Figure 5. As it was expected, all the five DNA repair mechanisms are sub-sets of DNA repair, given that these GO terms are child terms of DNA repair (Figure 4). This Venn diagram was created manually, because it exceeds the maximum number of elements supported for automated creation by Draw Venn; however the sub-sets were determined by using Draw Venn [23]. The contents of the Venn diagram are found in the Supplementary Information Table S1.

#### 3.1.3. *Arabidopsis thaliana* DNA Repair Genes

**Figure 6:**
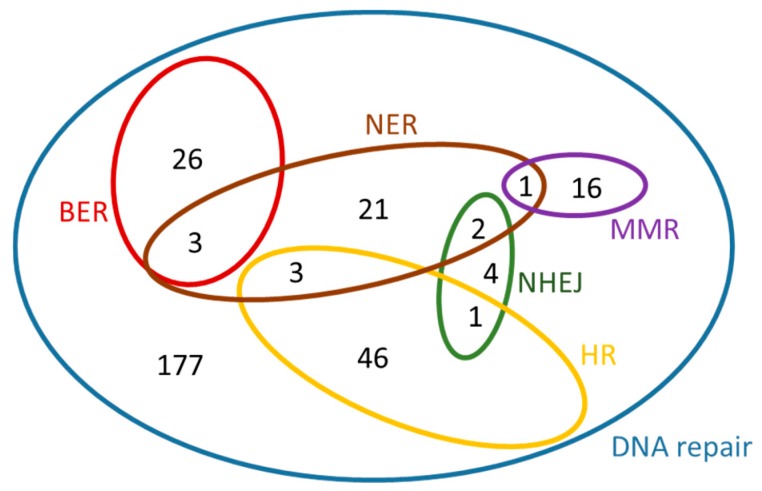
Venn diagram of the *Arabidopsis thaliana* genes that were found under each of the GO terms listed in Table 4.

In a similar manner, for *A. thaliana,* the dataset ‘*Arabidopsis thaliana* genes (TAIR10 (2010-09-TAIR10))’ was chosen from *Ensembl Plants* [22], *Biomart*, *Plant Mart*. For each search, a separate GO term listed in Table 4 was used as ‘Filter’. By selecting ‘Gene stable ID’, ‘Gene name’ and ‘RefSeq protein ID’ under ‘Attributes – Features’, six ‘.txt’ files were generated for *Arabidopsis thaliana.* A Venn diagram for these genes is presented in Figure 6. This diagram was created manually, because it exceeds the maximum number of elements supported for automated creation by the software used, however the sub-sets were determined using the *Draw Venn* application [22]. The contents of this diagram can be found in the Table S2 in Supplementary Information.

#### 3.1.4. Identification of Orthologies between *Homo sapiens* and *Arabidopsis thaliana* DNA Repair Genes

**Figure 7:**
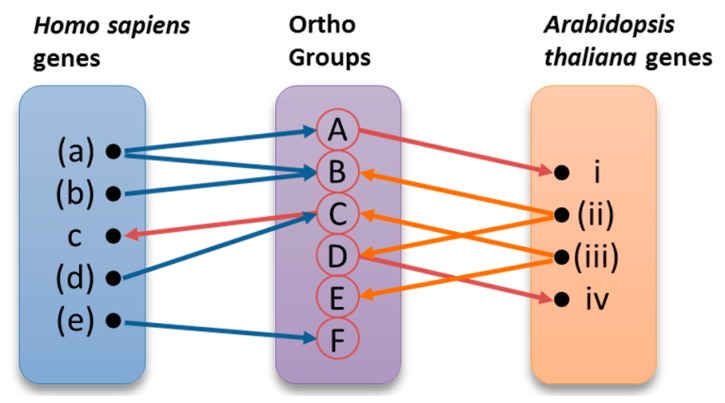
Comparative analysis strategy. The orthologues pairing process and the assignment of new roles to candidate genes are represented graphically. The genes (a), (b), (d), (e), (ii), and (iii) are already characterized, while c and i are new genes.

The derived twelve sets of genes were used as input to OrthoMCL-DB (http://orthomcl.cbil.upenn.edu) [29], providing for each gene a group of orthologues in the species under study. The results for the two species were collected and stored in a relational database. Data were combined in order to demonstrate known orthologies, propose new orthologies and, most importantly, suggest roles in DNA repair for orthologous genes. The orthologues pairing process, as well as the assignment of new roles to genes, are illustrated in Figure 7. Those of the initial genes that have been identified as annotated under the specific Gene Ontology (GO) terms are shown in parentheses. The same genes are represented in bold in the Supplementary Information (Tables S5–10), where the analytical results of this procedure can be found. Each one of these genes was used as input to Ortho MCL-DB, resulting to one or more Ortho Groups containing orthologous genes across several organisms. Ortho Groups A, B, and C contain both human and *Arabidopsis* genes. Ortho Groups D and E include only plant genes, while Ortho Group F contains only human genes. The overall procedure can be better described using the following example. As shown in Figure 7, the *H. sapiens* gene ‘(*a)’* belongs to A and B Ortho Groups. The *Arabidopsis* gene ‘*i*’ was identified in group A, by virtue of orthology, without having been previously annotated under the initial GO term. Ortho Group B contains also the human gene ‘(*a*)’ and *A. thaliana* gene ‘(*ii*)’. For this reason, the previously characterized genes ‘(*a*)’ and ‘(*ii*)’ were paired as orthologues. These kinds of pairs are presented in Table 5 and Table 6. By using the *A. thaliana* gene ‘(*ii)*’ as query in OrthoMCL-BD, we identified the orthogroup D, which also contains the not yet annotated *A. thaliana* gene ‘*iv*’. On the other hand, *H. sapiens* gene ‘(*e)’* belongs to group F, but since group F does not contain any *A. thaliana* gene, gene ‘(*e)’* was eventually excluded from the results.

Of note, protein names instead of gene names were used at this step. Therefore, for *Homo sapiens*, the ENSP (Ensembl protein ID) [7] and for *Arabidopsis thaliana*, the RefSeq (Reference Sequence) [8] nomenclature was used, respectively. For instance, the corresponding proteins of the gene *OGG1* (according to HGNC [9]) are ENSP00000305584 and NP_173590, in Human and *Arabidopsis thaliana*, respectively. Those DNA repair genes classified in orthologous groups are presented in Table 5, which contains condensed information presented in Table S5. The sets of orthologous genes between *Homo sapiens* (Hs) and *Arabidopsis thaliana* (At) retrieved for the five main DNA repair mechanisms are presented in Table 6.

### 3.2. ‘New Genes’ Emerging from Comparative Analysis

**Figure 8:**
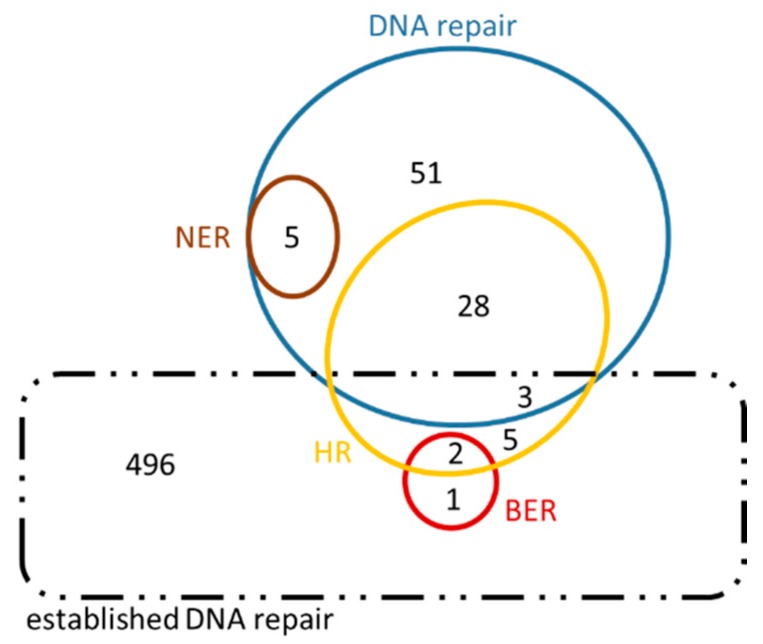
Venn diagram of *Homo sapiens* genes in their putative ‘new roles’. This diagram also contains an intersection of the genes in ‘new roles’ with ‘established DNA repair’ genes, in order to highlight the great number of genes arisen from this analysis that have not been previously characterized under the inclusive term ‘DNA repair’ (dashed black line).

**Figure 9:**
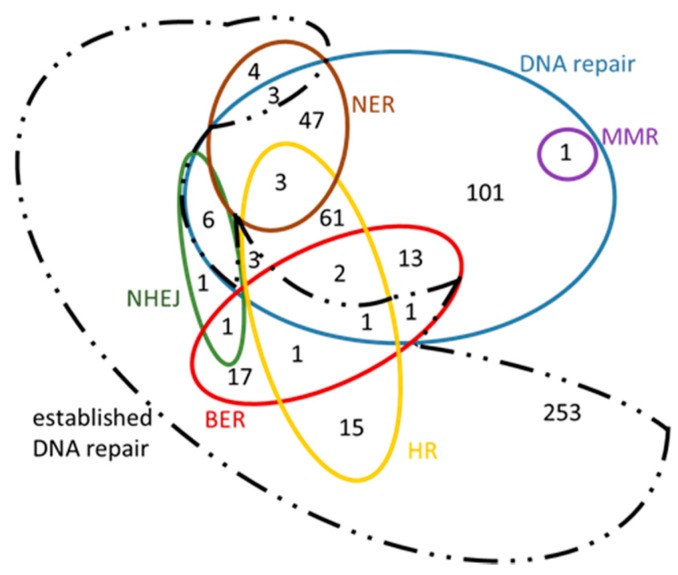
Venn diagram of *Arabidopsis thaliana* genes in their ‘new roles’. This diagram also contains the intersection with established DNA repair genes in order to highlight the great number of genes emerged from this analysis that have not been previously characterized under the inclusive term ‘DNA repair’ (dashed black line).

The sets of orthologous genes between *H. sapiens* and *A. thaliana* involved in the five main DNA repair mechanisms, retrieved as previously described, are reported in Table 6. Genes already known to participate in these mechanisms (termed as ‘old genes’) were included in Table 6. The bioinformatic analysis revealed a significant number of ‘new’ genes (e.g., ‘*c’*, ‘*i'* and ‘*iv’* genes described in Figure 7). Analytical results are available in Supplementary Information (Tables S5–10), where detailed lists of both ‘old’ and ‘new’ genes are provided. Possible relations are presented in the form of Venn diagrams (Figure 8 and Figure 9, Supplementary Information: Tables S3 and 4). Given that the newly retrieved genes (Table 7, columns 8 and 9) have not yet been characterized and assigned to those specific GO terms, these genes are considered as novel candidate players in DNA repair. These results imply that there are unexplored areas in *A. thaliana* for further research and discoveries. As shown in Table 7, 300 DNA repair genes are already known, whereas 243 ‘entirely new’ genes are suggested (see also Figure 9). Of those, 87 and 8 candidates are involved in the DSBs repair pathways HR and NHEJ, respectively. These ‘new genes’ could possibly play an auxiliary or parallel role in DSB repair, as recently discovered in the case of backup NHEJ [30]. Therefore, these genes could be described as ‘genes in new roles’.

Our results are presented in Venn diagrams (Figure 8 and Figure 9), in a concise manner, avoiding the repetition of the common genes among the mechanisms. For the sake of completeness, we have included in these diagrams the intersection with the initial DNA repair genes, referred to as ‘established DNA repair genes’ (black dashed line in Figure 8 and Figure 9). In these Venn diagrams, only the genes of Supplementary Information: Tables S5–S10 that are not in bold, i.e., those genes that have arisen from the present analysis, are included. Thus, it is not surprising that some genes are found outside the DNA repair set (blue line Figure 8 and Figure 9) and they therefore fall into the ‘established DNA repair’ group.

## 4. An In Vitro Approach Monitoring Key DNA Repair Genes

Germline variants in the human *BRCA1* gene are associated with familial breast and ovarian cancers [31]. The human BARD1 (BRCA1-associated RING domain protein 1) is essential for the sequestration of BRCA1 at DNA damage sites [32]. Moreover, the DDR-related protein PARP1 (Poly(ADP-ribose) polymerase-1) [33] was shown to mediate the function of BRCA1 in DDR [32]. Herein, the DNA-CL (crosslinks) comet assay, an assay modified for detection of DNA interstrand cross-links, was used to assess the effect of the *Arabidopsis thaliana* genes *BRCA1*, *BARD1* and *PARP1* on DNA damage repair. To this end, the alkaline/neutral (A/N) protocol of comet assay described in Angelis et al. [34], with the additional enzyme treatment step, was employed. Isolated nuclei from chopped *Arabidopsis thaliana* seedlings were embedded into a 0.7% agarose gel and lysed for 1 h. Then, the lysed nuclei were enzymatically treated. In particular, they were equilibrated for 20 min in a restriction endonuclease Sal1 buffer and then 50 μL of Sal1 solution (1U/ml) were added. Each gel was then spread on microscopic slides, covered with Parafilm and incubated in a sterile moist chamber for 50 min at 37 °C. The restriction enzyme digestion was stopped using TE buffer. Following enzyme treatment, the slides were dipped for 20 min into a DNA-unwinding solution (0.3 M NaOH; 10 mM EDTA), neutralized for 5 min in 1× TBE buffer and electrophoresed in the same buffer at 1 Volt/cm for 5 min.

**Figure 10:**
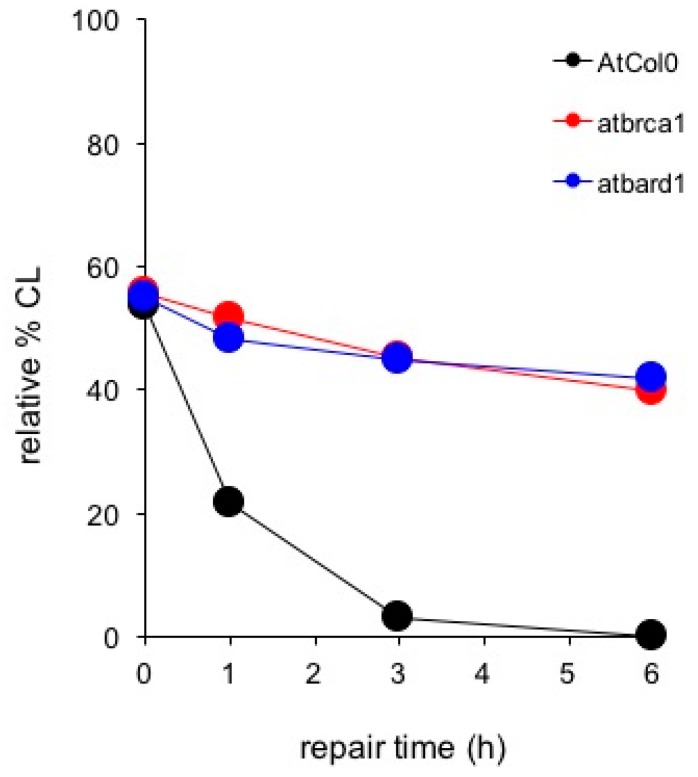
Comet assay depicting DNA damage repair in wild-type *Arabidopsis thaliana* (AtCol0) and the knockout DNA repair mutants *atbrca1* and *atbard1* following treatment with 200 μM Mitomycin C and post-treatment recovery for 1, 3 and 6 h.

**Figure 11:**
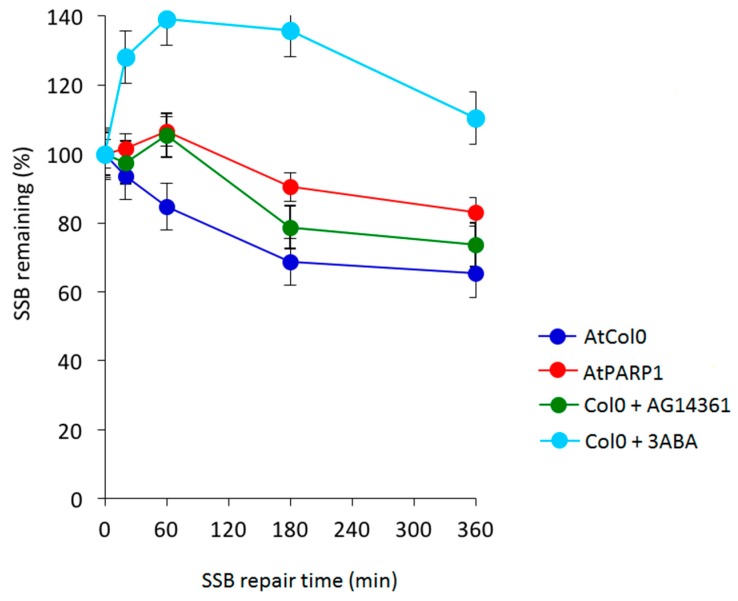
Effect of the knockdown mutation of AtPARP1 and of specific and broad spectrum inhibitors of PARP1 on SSB repair kinetics obtained by an A/N comet assay. SSBs generated following 1 hr treatment with 2 mM MMS (methyl methanesulfonate) in AtPARP1 and in *Arabidopsis* Col1, in the presence of 3 mM 3-aminobenzamide (3-ABA) and 10 μM HsPARP1 specific AG14361 inhibitors.

As shown in Figure 10, comparison of DDR kinetics revealed similarly reduced ability of the *AtBRCA1* and *AtBARD1* mutants to remove CL. Despite of the fact that wild type AtCol0 removed all CL after 3 h of DDR, residual DNA damage was observed in mutant *AtBRCA1*. The half-life of CL in *AtBRCA1* and *AtBARD1* (t_1/2_ = 10 h) is approximately 10 times longer compared to Col0 (t_1/2_ = 1.2 h). Therefore, *AtBRCA1* and *AtBARD1* can efficiently repair DNA interstrand cross-linked adducts generated by mitomycin C. On Figure 11 is shown the effect of SSB accumulation during the early stages of base excision repair (BER) recovery in *Arabidopsis* plants due to PARP1 impairment either by a knockout mutation leading to *AtPARP1* or by two PARP1 inhibitors, the selective PARP1 inhibitor AG14361, developed by Pfizer for the sensitization of human breast cancer cells prior to irradiation treatment, or the non-specific PARP inhibitor 3-aminobenzamide (3-ABA). Of interest, the PARP1-mediated signaling is conserved among kingdoms, since the selective AG14361 inhibitor of HsPARP1 is also effective in *Arabidopsis* and exhibits the same repair kinetic behavior as the knockout *AtPARP1* mutation, contrary to 3-ABA. The above observations lead to the suggestion that the genes *AtBRCA1*, *AtBARD1* and *AtPARP1* play an equally important role in DNA damage repair in plants, like *Arabidopsis thaliana*, as in animals and humans.

## 5. Conclusions

**Figure 12:**
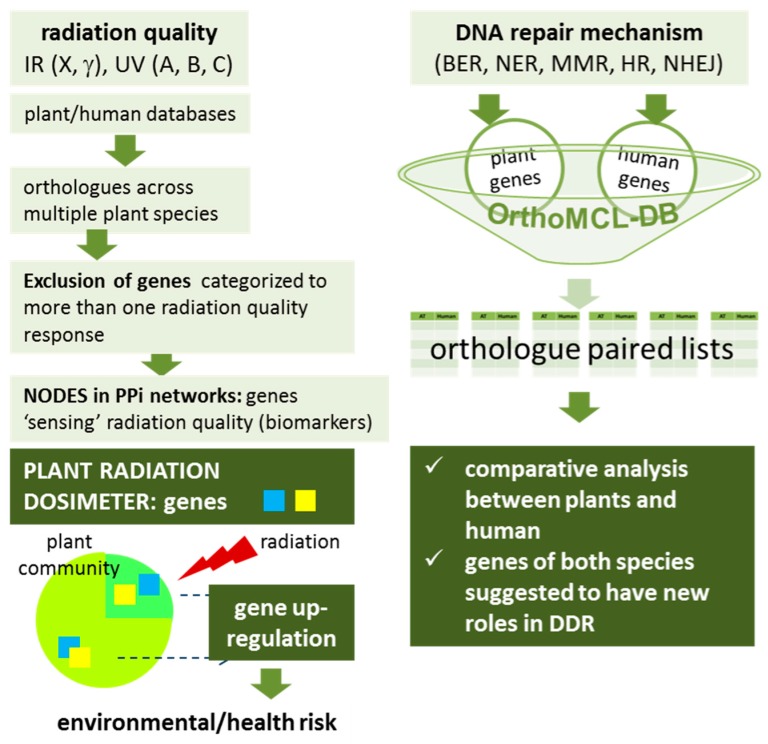
Schematic representation of the in silico methodology designed to select candidate genes for the plant radiation dosimeter as well as for the comparison of human and plant DNA repair machinery. BER, base excision repair. NER, nucleotide excision repair. MMR, mismatch repair. HR, homologous recombination. NHEJ, non-homologous end joining. DDR, DNA damage response. IR, ionizing radiation, UV, ultraviolet radiation. PPi, protein-protein interaction. Please see recent work by Pateras et al. [20], for analytical description of all DDR pathways.

The systematic bioinformatic approach employed in this study to select candidate genes for the ‘plant radiation dosimeter’ (Figure 12) revealed that, despite the fact that the last common ancestor of human and *A. thaliana* is traced approximately 1.5 billion years ago [35], fundamental mechanisms underlying the maintenance of genome integrity, as well as their associated genes, are conserved between animals and plants. In particular, we have identified plant genes with human counterparts that can be used as ‘signature radiation genes’ in order to allow direct comparisons on the primary mechanisms governing the DNA damage repair response to different types of radiation (ionizing and non-ionizing) across the tree of life. The expression patterns (up- or down-regulation) of the genes which have been classified in each part of the electromagnetic spectrum should be evaluated experimentally after exposure of the plant only to a certain component of the electromagnetic spectrum. It is expected that some of the proposed genes could actually be exploited as biomarkers of exposure to electromagnetic radiation, for assessing radiation risk in environment. The main conclusion is that the results from the in silico analysis performed herein are expected to provide the foundation for future research efforts in order to design a radiation biodosimeter. Apart from the REM biomarkers, a large number of putative genes suggested to participate in DDR was also identified in human and *Arabidopsis thaliana*. Our studies support the further development of a plant-based radiation biodosimeter. We believe, that the importance of using a low-cost non-animal system to monitor and estimate radiation risk is high since it helps in establishing a reliable methodology avoiding all the ethical issues associated in most cases with the use of animals or human cells. In addition, this plant-based platform maybe used in other cases for the screening of specialized drugs targeting for example DNA repair like PARP inhibitors, predict response to inhibitors of the DNA damage sensors ATM and ATR, and inhibitors of nonhomologous end joining etc. As recently discussed in Stover et al. [36], producing and validating reliable biomarkers will help boost the efficiency of DNA repair targeted therapies and exploit their role(s) on cancer treatment. Also in this case, plants may prove as a first-step screening tool on all of the above cases.

## Figures and Tables

**Table 1:** Selected Gene Ontology (GO) terms, their description and the component of the electromagnetic spectrum they represent.

GO Term	Annotation	Category
GO:0010212	response to ionizing radiation	Ionizing Radiation
GO:0010165	response to X-ray	X-ray
GO:0010332	response to gamma radiation	*γ*-ray
GO:0009411	response to UV	UV
GO:0070141	response to UV-A	UV-A
GO:0010224	response to UV-B	UV-B
GO:0010225	response to UV-C	UV-C

**Table 2:** Selected genes the products of which would serve as ‘exclusive’/highly specific biomarkers for the identification of radiation quality in planta, and therefore as specific indicators of the geographical region in which the ‘biosensor plant’ lives. These genes encode products that appeared as nodes of dense cliques of a protein-protein interaction network, created using STRING v10 (Figure 3). Genes are listed, along with a number, indicative of the multiplicity of their orthologues across the plant species under study. The symbol ‘#’ indicates the number of species.

γ-rays	#	X-rays	#	UV-A	#	UV-B	#	UV-C	#
RAD54	30	ATLIG4	28	AT2G21970.1	2	UVH3	32	TED4	3
AT4G14970	30	MIM	7			RPA70B	7	MC4	2
RPA1A	27	SMC6A	6			RPA70D	5	MC7	2
MSH5	23					XPB1	4	MC5	2
RAD51	18					XPB2	4	MC6	2
						AT3G48900	1	MC8	2
								HO4	1
								HO3	1
								ETL1	1
								PCC1	1
								PAD4	1

Abbreviations: ETL, Enhancer Trap Locus; LIG, Ligase; MIM, Missing In Metastases; MC, MicroCystin; MSH, MutS-Homolog; PAD, Protein Arginine Aeiminase; PCC, Pathogen and Circadian Controlled; RAD, RAdiation Sensitive; RPA, Replication Protein A; SMC, Structural Maintenance of Chromosome; UVH, UltraViolet Hypersensitive; XP, Xeroderma Pigmentosum; TED, reversal of the DET phenotype.

**Table 3:** Human orthologues of the resulting genes, proposed as exclusive biomarkers for the detection of the exposure to the several types of the electromagnetic spectrum.

Type of Radiation	*Arabidopsis thaliana*			Ortho Group	*Homo Sapiens*		
	TAIR	Gene Name	RefSeq		ENSP	HGNC	GO
**γ-rays**	RAD54	AT3G19210	NP_188552	OG5_127098	ENSP00000336606	RAD54B	GO:0010212
					ENSP00000396113	RAD54L	GO:0010212
		AT4G14970	NP_193233	OG5_132711	ENSP00000287647	FANCD2	GO:0010332
	RPA1A	AT2G06510	NP_973433	OG5_127539	ENSP00000254719	RPA1	--
	MSH5	AT3G20475	NP_188683	OG5_129379	ENSP00000364894	MSH5	--
					ENSP00000387668	MSH5	
					ENSP00000394619	MSH5	
					ENSP00000394649	MSH5	
					ENSP00000406868	MSH5	
					ENSP00000407047	MSH5	
					ENSP00000409207	MSH5	
	RAD51	AT1G07745	NP_172254	OG5_132909	ENSP00000378090	RAD51D	GO:0010212
**X-rays**	ATLIG4	AT5G57160	NP_568851	OG5_130132	ENSP00000402030	LIG4	GO:0010212 GO:0010165 GO:0010332
	MIM	AT5G61460	NP_200954	OG5_127751	ENSP00000370672	SMC6	GO:0010165
	SMC6A	AT5G07660	NP_196383	OG5_127751	ENSP00000370672	SMC6	--
**UV-A**	SEP2	AT2G21970	NP_565524	OG5_178242	no	--	--
**UV-B**	UVH3	AT3G28030	NP_566830	OG5_128675	ENSP00000347978	ERCC5	GO:0009411 GO:0010225
	RPA70B	AT5G08020	NP_196419	OG5_127539	ENSP00000254719	RPA1	--
	RPA70D	AT5G61000	NP_200908	OG5_127539	ENSP00000254719	RPA1	--
	XPB2	AT5G41360	NP_568591	OG5_127208	ENSP00000285398	ERCC3	GO:0009411
	XPB1	AT5G41370	NP_568592	OG5_127208	ENSP00000285398	ERCC3	--
	GEN2	AT3G48900	NP_001118795	OG5_174560	no	--	--
**UV-C**	TED4	AT2G26670	NP_001118392	OG5_140322	no	--	--
	MC4	AT1G79340	NP_178052	OG5_147205	no	--	--
	MC8	AT1G16420	NP_173092	OG5_134790	no	--	--
	MC7	AT1G79310	NP_178049	0		--	--
	MC5	AT1G79330	NP_178051	OG5_147205	no	--	--
	MC6	AT1G79320	NP_178050	0		--	--
	HO4	AT1G58300	NP_176126	OG5_140322	no	--	--
	HO3	AT1G69720	NP_177130	OG5_140322	no	--	--
	ETL1	AT2G02090	NP_178318	OG5_129286	ENSP00000351947	SMARCAD1	--
	PCC1	AT3G22231	NP_566702	OG5_144902	no	--	--
	PAD4	AT3G52430	NP_190811	OG5_190312	no	--	--

**Table 4:** Gene Ontology (GO) terms describing DNA repair pathways.

GO Term	Annotation	Abbreviation
GO:0006281	DNA repair	DNA repair
GO:0006284	base-excision repair	BER
GO:0006289	nucleotide-excision repair	NER
GO:0006298	mismatch repair	MMR
GO:0000724	double-strand break repair via homologous recombination	HR
GO:0006303	double-strand break repair via non-homologous end joining	NHEJ

**Table 5:** DNA repair groups of orthologous genes between *Homo sapiens* and *Arabidopsis thaliana*. This table contains only the initial genes from the two organisms that have been already characterized under the GO term ‘DNA repair’ and not the genes that have arisen from this analysis.

*Homo sapiens*	*Arabidopsis thaliana*	*Homo sapiens*	*Arabidopsis thaliana*	*Homo sapiens*	*Arabidopsis thaliana*	*Homo sapiens*	*Arabidopsis thaliana*
ERCC6	CHR8	GINS4	SLD5	GTF2H4	AT4G17020	ALKBH3	ALKBH2
NSMCE2	MMS21	UNG	ATUNG	DCLRE1A	SNM1	XRCC1	XRCC1
RNASEH2A	AT2G25100	APTX	BHLH140	XRCC5	KU80	XRCC2	XRCC2
TOP3A	TOP3A	ALKBH1	AT1G11780	NSMCE1	emb1379	XRCC4	XRCC4
RECQL RECQL5 WRN BLM	RECQL4A MED34	RAD23A RAD23B	RAD23A RAD23B RAD23C RAD23D	RPA1	RPA1A RPA1B RPA1C RPA1D	ERCC5	UVH3
						OGG1	OGG1
						ERCC6L2	SWI2
						ERCC1	ERCC1
KAT5	HAM2 HAM1	DDB1	DDB1A DDB1B	KIF22	AT5G02370	ASF1A	ASF1A ASF1B
				MRE11A	MRE11		
RAD51C	RAD51C	XRCC3	XRCC3	DDB2	DDB2	GEN1	GEN1
EXO1	AT1G18090	MPG	MAG	RAD1	AT4G17760	REV3L	REV3
APEX2	ARP APE1L APE2	UBE2V1	UEV1A UEV1B UEV1C	RPS3	RPS3A RPS3B RPS3C	UBE2A UBE2B	UBC1 UBC2 UBC3
MLH1	MLH1	SHFM1	ATDSS1(V)	POLD3	POLD3	XPC	ATRAD4
MTOR	TOR	SSRP1	SSRP1	XRCC6	KU70	ZSWIM7	AT4G33925
CUL4ACUL4B	CUL4	DMAP1	SWC4	TDP1	TDP1	ERCC4	UVH1
		MCM8	MCM8	NSMCE4A	NSE4A	ERCC2	UVH6
RRM2B	RNR2A TSO2	SLX1A SLX1B	AT2G30350	DMC1 RAD51	DMC1 RAD51	ERCC3	XPB2 XPB1
CDC5L	CDC5	ACTL6A	ARP4	PARP2	PARP2	BRCA2	BRCA2B
PMS2	PMS1	PNKP	ZDP	GINS2	GINS2	POLL	AT1G10520
SUPT16H	SPT16	ATR	ATR	POLH	POLH	FANCL	AT5G65740
MSH6	MSH6 MSH7	RAD9A RAD9B	RAD9	PCNA	PCNA PCNA2	LIG1	LIG1 AT1G49250
NEIL2	FPG1	CHAF1A	FAS1	CDC45	CDC45	MSH2	MSH2
NTHL1	NTH1 NTH2	POLR21	NRPB9A NRPB9B	GTF2H2 GTF2H2C	ATGTF2H2	RPA2	RPA2A RPA2B
MUTYH	MYH	GTF2H1	TFB1-1	INO80	INO80	CHAF1B	FAS2
PRPF19	PRP19A	LIG4	LIG4	MSH3	MSH3	SMC5	SMC5
FEN1	FEN1	RFC1	RFC1	GTF2H3	AT1G18340	SMARCAD1	ETL1
RAD17	RAD17	PARP1	PARP1				

**Table 6:** Sets of retrieved orthologous genes between *Homo sapiens* (Hs) and *Arabidopsis thaliana* (At) suggested to be implicated in the five main DNA repair mechanisms. For GO terms refer to Table 4. BER, base excision repair; NER, nucleotide excision repair; MMR, mismatch repair; HR, homologous recombination; NHEJ, non-homologous end joining.

BER		NER		MMR		HR		NHEJ	
*Hs*	*At*	*Hs*	*At*	*Hs*	*At*	*Hs*	*At*	*Hs*	*At*
APEX2	ARP APE1L APE2	ERCC3	XPB2 XPB1	MSH6	MSH7 MSH6	WRN BLM	RECQL4A	PARP2 PARP1	PARP2
NTHL1	NTH2 NTH1	RAD23B RAD23A	RAD23A RAD23B RAD23C RAD23D	RNASEH2A	AT2G25100	RAD51 DMC1	RAD51	XRCC6	KU70
UNG	ATUNG	ERCC2	UVH6	MLH1	MLH1	FIGNL1	AT3G27120	XRCC5	KU80
FEN1	FEN1	GTF2H2 GTF2H2C	ATGTF2H2	PCNA	PCNA PCNA2	RAD54L RAD54B	CHR25	XRCC1	XRCC1
MRE11A	MRE11	POLR2I	NRPB9A NRPB9B	MSH2	MSH2	MTOR	TOR		
OGG1	OGG1	XPC	ATRAD4	PMS2	PMS1	ERCC4	UVH1		
MUTYH	MYH	ERCC5	UVH3	MSH5	MSH5	ATR	ATR		
MPG	MAG	GTF2H4	AT4G17020	MSH4	MSH4	GINS2	GINS2		
NEIL2	FPG1	GTF2H3	AT1G18340	MSH3	MSH3	SMC5	SMC5		
		DDB1	DDB1A DDB1B	MLH3	MLH3	ERCC1	ERCC1		
		GTF2H1	TFB1-1 TFB1-3			GINS4	SLD5		
		LIG4	LIG4			MCM8	MCM8		
		POLL	AT1G10520			MUS81	MUS81		
						CDC45	CDC45		
						NSMCE1	emb1379		
						SHFM1	ATDSS1(V)		
						POLD3	POLD3		
						XRCC3	XRCC3		
						NSMCE2	MMS21		
						BRCA2	BRCA2A BRCA2B		
						NBN	NBS1		
						XRCC2	XRCC2		
						ZSWIM7	AT4G33925		
						RAD51B	RAD51B		

Abbreviations: APE, Apurinic/Apyrimidinic Endonuclease; ARP, Apurinic Endonuclease-Redox protein; AT, *Arabidopsis thaliana*; ATR, Ataxia Telangiectasia and Rad3 Related; BLM, Bloom Syndrome RecQ like Helicase; BRCA, Breast Cancer Protein; CHR, Chromatin Remodeller; CDC, Cell Division Cycle; DDB, DNA Damage Binding Protein; DMC, Disrupted Meiotic cDNA; DSS1, Deleted in Split-Hand/Split Foot Syndrome; ERCC, Excision Repair Cross-Complementing Protein; FEN, Flap Endonuclease; FIGNL, Fidgetin-Like Protein; FPG, Formamidopyrimidine-DNA Glycosylase; GTF2H, synonimous of ERCC3; GINS, Go-Ichi-Ni-San; LIG, Ligase; MAG, Myelin-Associated Glycoprotein; MCM, Minichromosome Maintenance; MLH, MutL-homolog; MMS, Methyl Methanesulfonate Sensitive; MPG, 3-Methyladenine-DNA Glycosylase; MRE, Meiotic Recombination 11 homolog; MSH, MutS-homolog; MTOR, Mammalian Target of Rapamycin; MUS81, Structure-Specific Endonuclease Subunit; MUTYH and MYH, MuY DNA Glycosylase; NBN, Nibrin; NBS, Nijmegen Breakage Syndrome; NEIL, eukaryotic homolog of *Escherichia coli* endonuclese VIII (Nei); NRP, Nuclear RNA Polymerase; NSMCE, Non-smc Element 2 Mms21 homolog; NTHL, human homolog of NTH1; NTH, endonuclease III from *E. coli*; OGG1, 8-Oxoguanine DNA Glycosylase; PARP, Poly (ADP-ribose) Polymerase; PCNA, Proliferating Cell Nuclear Antigen; PMS, Postmeiotic Segregation Increased; RAD, Radiation Sensitive; REC, Recombinant; SLD, Synthetic Lethality with dpb11; SHF, MutS; SMC, Structural Maintenance of Chromosome; TFB, TATA-Binding Protein; TOR, Target of Rapamycin; UNG, Uracil DNA Glycosylase; UVH, Ultraviolet Hypersensitive; XRCC, X-Ray Repair Cross-Complementing; ZSWIM, Zinc Finger SWIM-Domain Containing Protein; WRN, Werner.

**Table 7:** Quantitative results obtained from the comparative analysis of DNA repair mechanisms in plants and humans. In the first column, the various ‘lemmas’ (annotations) for DNA repair and its subpathways are presented, accompanied with the corresponding GO terms. The 2nd column is referred to the number of the already characterized *Homo sapiens* (Hs) genes. The 3rd refers to those genes of the 2nd column that have orthologues (one or more) in *Arabidopsis thaliana* (At). The 4th column is just the percentage (those that have orthologues/all genes). Correspondingly, 5th–7th columns refer to *Arabidopsis thaliana*. Underlined (8th–9th column) are the ‘new’ genes identified from our analysis for Hsa and Atha respectively. BER, base excision repair. NER, nucleotide excision repair. MMR, mismatch repair. HR, homologous recombination. NHEJ, non-homologous end joining.

1. Mechanism (GO Term)	2. # *Hs* Genes	3. # *Hs* Genes That Have Orthologues in *At*	4. %	5. # *At* Genes	6. # *At* Genes That Have Orthologues in *Hs*	7. %	8. # Suggested Genes Arisen from Our Analysis for *Hs*	9. # Suggested Genes Arisen from Our Analysis for *At*
**DNA repair** **GO:0006281**	507	259	51.1	300	185	61.7	86	243
**BER** **GO:0006284**	52	32	61.5	29	12	41.4	3	36
**NER** **GO:0006289**	124	59	47.6	30	21	70.0	5	57
**MMR** **GO:0006298**	43	14	32.6	17	12	70.6	0	1
**HR** **GO:0000724**	162	76	46.9	50	37	74.0	38	87
**NHEJ** **GO:0006303**	73	9	12.3	7	4	57.1	0	8
**Total number of ‘DNA repair’ genes associated with “new roles” arisen from this analysis (see Figure 8 and Figure 9)**							95	281
**Total number of “entirely new” genes that have arisen from this analysis after the comparison with the “previously established” DNA repair genes**							84	234

## Supplementary Materials

The following are available online at http://www.mdpi.com/2072-6694/9/6/65/s1. Table S1: Human DNA repair genes. Contents of the Venn diagram presented in Figure 5 in the main text, Table S2: Arabidopsis thaliana DNA repair genes. Contents of the Venn diagram presented in Figure 6 in the main text, Table S3: *Homo sapiens* genes that stemmed from the present analysis (i.e., contents of the Venn diagram shown in Figure 8 in the main text), Table S4: Arabidopsis thaliana genes that stemmed from the present analysis (i.e., contents of the Venn diagram shown in Figure 9 in the main text), Table S5. DNA repair genes in human and Arabidopsis thaliana, grouped according to orthology, Table S6: BER genes in human and Arabidopsis thaliana, grouped according to orthology, Table S7: NER genes in human and Arabidopsis thaliana, grouped according to orthology, Table S8: MMR genes in human and Arabidopsis thaliana, grouped according to orthology, Table S9. HR genes in human and Arabidopsis thaliana, grouped according to orthology, Table S10: NHEJ genes in human and Arabidopsis thaliana, grouped according to orthology.

## Abbreviations

DDR	DNA damage response
SSB	single-strand breaks
DSB	double-strand breaks
NER	nucleotide excision repair
BER	base excision repair
MMR	Mismatch repair
HR	homologous recombination
NHEJ	non-homologous end joining
UV	ultraviolet radiation
IR	ionizing radiation
PAXX	PAralog of XRCC4 and XLF
REM	radiation exposure monitoring
