# Supplementary material for: Bridging Plant and Human Radiation Response and DNA Repair through an In Silico Approach
Source: Cancers (Basel). 2017 Jun 6;9(6):65. doi: 10.3390/cancers9060065 (PMC5483884; doi:10.3390/cancers9060065)
Supplement: Supplementary file 1 [file cancers-09-00065-s001.pdf]

Supplementary Information: Bridging Plant and Human Radiation Response and DNA Repair through an *in silico* Approach

Zacharenia Nikitaki, Athanasia Pavlopoulou, Marcela Holá, Mattia Donà, Ioannis Michalopoulos, Alma Balestrazzi, Karel J. Angelis and Alexandros G. Georgakilas

Table S1. Human DNA repair genes. Contents of the Venn diagram presented in Figure 5 in the main text.

**BER ∩ NER ∩ HR ∩ MMR ∩ DNA repair:** RPA2 RPA1 RPA3
**BER ∩ NER ∩ HR ∩ NHEJ ∩ DNA repair:** LIG3
**BER ∩ NER ∩ MMR ∩ DNA repair:** POLD2 PCNA POLD1 POLD3 LIG1 POLD4 HMGB1
**BER ∩ NER ∩ HR ∩ DNA repair:** RFC2 RFC3 XRCC1 RFC4 RFC5 PARP1
**BER ∩ HR ∩ NHEJ ∩ DNA repair:** POLQ
**NER ∩ HR ∩ NHEJ ∩ DNA repair:** SUMO1 UBE2V2 UBE2N
**BER ∩ NER ∩ DNA repair:** TP53 PNKP NEIL2 NEIL1 NTHL1 NEIL3 RFC1 OGG1 ERCC6 POLE
**BER ∩ MMR ∩ DNA repair:** TDG MUTYH
**BER ∩ HR ∩ DNA repair**: SIRT6 DNA2 FEN1 WRN
**BER ∩ NHEJ ∩ DNA repair:** HMGA2
**NER ∩ MMR ∩ DNA repair:** XPC
**NER ∩ HR ∩ DNA repair:** HUS1 FAN1 UBB RAD51D ERCC1 BRCA2 ERCC4 RAD52 SUMO2 SLX4 UBE2I HUS1B UBA52 RBBP8 RPS27A UBC
**NER ∩ NHEJ ∩ DNA repair:** POLL LIG4 PRPF19
**MMR ∩ HR ∩ DNA repair:** EXO1 ENSG00000282827 ABL1
**MMR ∩ NHEJ ∩ DNA repair:** MLH1
**HR ∩ NHEJ ∩ DNA repair:** UIMC1 HIST1H4I HIST1H4B NBN HIST4H4 MDC1 HIST1H4J KDM4D BRCC3 HIST1H4E HIST1H4L HERC2 BRCA1 PIAS4 HIST1H4H BARD1 WHSC1 BABAM1 HIST3H3 HIST2H4A RNF8 ATM HIST1H4D RNF168 HIST2H4B KAT5 TP53BP1 HIST1H4A H2AFX HIST1H4C HIST1H4F RAD50 HIST1H4K MRE11A PSMD14 FAM175A BRE
**BER ∩ DNA repair:** PARG CCNO USP47 ENSG00000268361 MBD4 SMUG1 APEX2 PARP2 PRMT6 APEX1 POLG HMGA1 ENSG00000254469 MPG UNG POLB HUWE1
**NER ∩ DNA repair:** MMS19 SLC30A9 ERCC5 POLR2G COPS6 POLR2L POLK MNAT1 CUL4B DDB1 EP300 XAB2 BIVM-ERCC5 RPA4 COPS7A COPS2 HMGN1 SUMO3 ZNF830 GTF2H1 COPS5 RAD23A CETN2 GTF2H2C CUL4A GTF2H5 GPS1 POLR2C DDB2 ATXN3 POLR2K SIRT1 RAD23B GTF2H2 PPIE RBX1 POLR2F GTF2H4 ERCC8 POLR2B AQR USP45 USP7 COPS4 XPA POLR2I COPS8 POLR2J POLR2E ERCC3 GTF2H3 UVSSA CDK7 COPS3 CHD1L TCEA1 RNF111 POLR2D POLR2H ERCC2 GTF2H2C_2 CCNH POLR2A ISY1 COPS7B

Table S1. *Cont.*

**MMR ∩ DNA repair:** RNASEH2A MLH3 SETD2 PRKCG MSH3 MSH5-SAPCD1 MSH5 MSH4 AXIN2 MSH6 PMS1 TREX1 TP73 MSH2 PMS2
**HR ∩ DNA repair:** YY1 TIPIN ZSWIM7 AP5Z1 RTEL1 KDM1A SMC5 CLSPN RAD1 RMI1 EME1 PPP4R2 TOPBP1 RHNO1 TOP3A RAD51 RAD51B MMS22L BLM NSMCE2 SLX1B DMC1 XRCC3 SFPQ GEN1 WDR48 RAD54B TERF2IP PARPBP BRIP1 RAD9B PALB2 SPIDR RAD51C SWI5 GINS4 SFR1 MEIOB RNF4 MCM9 FIGNL1 AP5S1 CHEK1 FBXO18 NABP2 TONSL MORF4L1 RAD9A INO80 RTEL1-TNFRSF6B ATR ATRIP REC8 MUS81 RAD54L CDC45 SWSAP1 MCM8 FIGN SLX1A RMI2 POLN SHFM1 TEX15 CCDC155 ZFYVE26 CDC7 GINS2 NSMCE1 PPP4C CDK2 NABP1 SMC6 TIMELESS RAD21L1 HELQ RAD17 RAD51AP1 XRCC2
**NHEJ ∩ DNA repair:** POLA1 PAXIP1 C9orf142 RIF1 UVRAG XRCC4 XRCC5 PRKDC DCLRE1A DEK POLM XRCC6 NHEJ1 KDM2A SETMAR XRCC6BP1 DCLRE1B DCLRE1C
**DNA repair:** KDM4A ESCO2 DMAP1 NSMCE3 MTOR BTG2 NPAS2 DDX1 ATRX KIN CDKN2D INO80E SLF1 UBR5 SSRP1 GGN IGHMBP2 STUB1 UBE2A HIST3H2A ENSG00000269307 BACH1 EYA1 CHAF1A FAAP100 DTL TFPT ENDOV OTUB1 MORF4L2 TREX2 RFWD3 PRIMPOL NSMCE4A NFRKB MEN1 INO80B FANCA EEPD1 HLTF RECQL POLI CIB1 SMARCA5 PML RPS27L POLG2 BCCIP INIP CSNK1E CDK9 CDC14B MCRS1 NONO PARP3 CHAF1B ACTR8 REV3L TAOK3 PPP5C STRA13 ASCC3 TIGAR ACTL6A TTC5 ALKBH1 TERF2 WDR33 BAZ1B RRM2B HINFP GADD45A RUVBL1 SPATA22 SMC3 KPNA2 NUDT1 RCHY1 FTO EPC2 EYA4 UBE2F ASCC2 UBE2D3 SMARCAD1 RBM14 SHPRH RAD21 REV1 UCHL5 EME2 EGFR UBE2U ALKBH2 ZRANB3 KIAA0430 ZBTB1 PARP4 ASF1A PAPD7 FOXM1 UBE2V1 CEP164 TICRR JMY POLE2 USP43 FANCM HSPA1A WRNIP1 UBA7 SETX CDK1 MC1R PIF1 CHRNA4 FANCE SMARCB1 KIF22 CBX8 DTX3L ASCC1 APTX CDC5L CSNK1D EYA3 BOD1L1 MGME1 EXO5 FBXO6 RPAIN TNP1 CINP FANCL SPRTN UHRF1 APLF NPM1 MAPK8 KIAA0101 APBB1 USP10 ALKBH3 RPS3 RAD18 APITD1 CEBPG USP28 SUPT16H FANCI UBE2B PARP9 TRIP12 RUVBL2 FAAP24 INO80D FANCF TRIM25 TAOK1 UFD1L MGMT MUM1 VCP ENSG00000267618 SMG1 ZBTB32 KDM4B TWIST1 SLF2 CHEK2 NUDT16 TDP2 FANCC INO80C ERCC6L2 TRRAP SMC1A SYCP1 TRIP13 EID3 NCOA6 MAD2L2 UBE2L6 PTTG1 USP1 NPLOC4 UPF1 PSME4 EMSY HMGB2 TRIM28 FANCB CDCA5 UBE2T TMEM161A RECQL5 UBE2W ACTR5 MTA1 INTS3 USP3 ISG15 FANCG TDP1 EYA2 RDM1 FGF10 FANCD2 FAAP20 TEX12 ASTE1 POLH RNF169 FZR1

Table S2. *Arabidopsis thaliana* DNA repair genes. Contents of the Venn diagram presented in Figure 6 in the main text.

**BER ∩ NER ∩ DNA repair:** MRE11 OGG1 FPG1
**MMR ∩ NER ∩ DNA repair:** ATRAD4
**NER ∩ HR ∩ DNA repair:** ERCC1 UVH1 AT1G52530
**BER ∩ NHEJ ∩ DNA repair:** AT1G10520 LIG4
**HR ∩ NHEJ ∩ DNA repair:** ATR
**BER ∩ DNA repair:** AT1G19480 MYH AT1G80850 AT5G57970 ATUNG NTH2 AT3G50880 AT1G15970 DML3 MAG AT3G12710 AT3G47830 NTH1 APE2 AT1G75090 AT1G75230 FEN1 AT3G07930 ARP DML2 DME AT2G10550 APE1L ROS1 AT1G13635 AT5G44680
**NER ∩ DNA repair:** XPB2 AT1G62886 AT1G18340 AT1G12400 AT4G17020 RAD23C UVH3 UVH6 RAD23A DDB1B TFB1-3 ATGTF2H2 RAD23B DDB1A TFB1-1 AT5G16090 PNG1 NRPB9B XPB1 NRPB9A RAD23D
**MMR ∩ DNA repair:** MLH3 MSH7 AT1G65070 MSH3 AT3G15960 PCNA PCNA2 MSH5 MSH4 AT2G25100 AT5G54090 MSH6 PMS1 MLH1 MSH1 MSH2
**HR ∩ DNA repair:** ORC3 SDS SMC5 emb1379 AT3G15550 RMI1 RAD51 RAD51B FAS2 CHR25 SNI1 MMS21 RECQL4A AT4G33925 XRCC3 BRCA1 RAD5 AT3G27120 BARD1 ATDSS1(V) ASF1A FAS1 SLD5 AT2G03430 BRCA2B AT1G59760 AT3G42860 POLD3 ASF1B AT5G47870 DMS3 MUS81 CDC45 MCM8 AT1G71310 AT4G16970 BRCA2A POLA2 SMC6B NBS1 SMC6A GINS2 AT3G48210 CTF7 TOR XRCC2
**NHEJ ∩ DNA repair:** KU80 PARP2 XRCC1 KU70
**DNA repair:** AT4G32970 AT5G02370 MED34 CHC1 NSE4B RPA3B DET1 RPA2B AT2G01440 AT1G51210 ATRX AT2G02550 WHY2 SSRP1 PA200 RPA1B ATCSA-1 POLIA AT5G05670 GEN2 UVR3 AT5G49110 PHR1 HAM1 ASY1 MAPR4 DRT102 PRP19A AT3G51700 SNM1 RPS3C ASHH1 REV3 TOP3A RAD51D NET1D AT5G43530 BOLA2 AT2G19490 UEV1A DMC1 XRCC4 PARP3 REV7 UBC3 RNR2A RECA3 GEN1 GPAT9 NSE4A AT5G15610 EXO1 CHR8 AT1G12244 CRY2 DRT101 NAP1;1 TRFL10 EME1B AT1G11240 RPA1E HAM2 SMC3 TSO2 AT1G18090 AT1G49250 AT4G14970 UBC1 PRP19B ARP4 DSE1 AT3G13226

MSI4 OFP1 RAD51C DDB2 BRCC36A REV1 AT5G23910 ALKBH2 ATDPB2 ARP4A MND1 AT2G30350 POL2B RPA1D RECQL4B SWI2 AT3G52905 AXR1 XRI1 AT1G77620 WHY1 RPS3A MCM9 AT5G28780 ETL1 ZDP AT1G05840 AT2G23080 CRY3 AT4G31150 CML19 AT3G02400 AT1G14340 TTN8 AT1G78790 CUL4 AT3G11100 SPT16 UBC35 ETG1 RPA1A ATSRP3 RPA2A BRCC36B INO80 RECA AT4G30825 DUF9 CAPH2 AT5G07400 RFC1 AT3G04620 UEV1C EME1A AT5G50340 TAF14B MER3 AT3G51690 RPA1C AT1G49980 RAD50 AT5G65740 POLIB LIG1 AT1G61410 UBC28 PHR2 RECQSIM CDC5 AT3G12210 RPA3A NAP1;3 DUT AT4G17760 DRT111 SMC4 WHY3 POL2A AT3G09100 UEV1B ATSRP2 RAD9 AT1G11780 SWC4 AT1G02160 ARP5 DRT100 COP1 GR1 CRY1 AT4G32960 NAP1;2 TDP1 MPC1 UBC2 BHLH140 AtLIG6 AT4G30820 AT3G32920 RAD17 PARP1 SYN2 AT2G43280 RPS3B POLH

Table S3. *Homo sapiens* genes that stemmed from the present analysis (i.e. contents of the Venn diagram shown in Figure 8 in the main text).

Name	#	Genes
HR ∩ DNA repair ∩ established DNA repair	3	HLTF SMC6 SUMO3
BER ∩ HR ∩ established DNA repair	2	RECQL RECQL5
BER ∩ established DNA repair	1	BLM
HR ∩ established DNA repair	5	ESCO2 HIST3H2A CHAF1A CHAF1B ASF1A
NER ∩ DNA repair	5	NGLY1 CETN1 CREBBP ISY1-RAB43 POLR3B
HR ∩ DNA repair	28	HIST1H2AA TTF2 HIST1H2AM HIST1H2AJ HIST1H2AE HIST1H2AD SKIV2L2 ASF1B ANKRD28 ESCO1 ANKRD44 HIST1H2AC SPC25 HIST2H2AA3 ANKRD52 HIST1H2AH CDK5 BOLA3 KAT8 HIST2H2AB HIST1H2AG HIST1H2AI YY2 POLA2 KAT7 HIST2H2AC BOLA2B SPAST
DNA repair	51	HORMAD2 SMARCD3 DNA repair CSNK1A1L HMGB3 PAPD5 ACTL6B SMARCD1 YEATS4 UBA1 CRY2 RFWD2 NOL12 MACROD1 RBM17 SMARCD2 NAE1 SERPINI1 MND1 GPAT4 SERPINB4 UBA6 SERPINB1 ASIC2 SMC1B RRM2 EIF3M SRPRB DUT GNB1L TMEM189-UBE2V1 CRY1 MPC1 DET1 BOLA2 UBE2D4 ASIC1 CYB5D2 CSNK2A2 CDC20 SERPINB3 EPC1 MCMBP OTUB2 CSNK1A1 SMARCAL1 GPAT3 SMARCA1 UBE2D1 NAP1L1 RNGTT
Established DNA repair	492	-not presented-

Table S4. *Arabidopsis thaliana* genes that stemmed from the present analysis (i.e. contents of the Venn diagram shown in Figure 9 in the main text).

Names	#	Elements
BER∩DNA repair ∩ HR ∩ established DNA repair	1	RECQL4B
BER ∩ DNA repair ∩ established DNA repair	1	RPA1E
NER ∩ DNA repair ∩ established DNA repair	3	PRP19B ATCSA-1 AT1G49980
HR ∩ DNA repair ∩ established DNA repair	3	MCM9 RAD51D EXO1
BER ∩ HR ∩ established DNA repair	1	MED34

Table S4. *Cont.*

BER ∩ NHEJ ∩ established DNA repair	1	PARP1
BER ∩ HR ∩ DNA repair	2	RECQL1 RECQL3
NER ∩ HR ∩ DNA repair	3	SUMO1 SUMO2 SUMO8
BER ∩ established DNA repair	17	PCNA2 CHR8 AT1G49250 RPA1D RPA1A RFC1 RPA1C LIG1 RPA2B RPA1B RECQL4A PCNA PARP2 ZDP POLD3 XRCC1 RPA2A
NER ∩ established DNA repair	4	PRP19A SNM1 DDB2 CUL4
HR ∩ established DNA repair	15	HAM1 TOP3A DMC1 GEN1 HAM2 AT2G30350 AT4G17760 RAD17 AT5G43530 BOLA2 AT1G18090 RAD51C UBC35 INO80 RAD9
NHEJ ∩ established DNA repair	1	XRCC4
BER ∩ DNA repair	13	RFC2 RFC3 PARG2 POLD2 SRT1 PARG1 STI PRMT6 emb2411 RFC4 RFC5 POLD4 F14L17.24
NER ∩ DNA repair	47	HAC5 RBX1A NRPB2 AT2G45700 CSN3 EMB2765 CDKD-1 AT3G02860 RBX1B CYCH1-1 NRPB3 CSN4 CDKD-3 NRPB10 CSN5A NRPB10L AT3G18790 NRPD7 HAC12 AT1G27420/F17L21.21 HAC04 HAC2 UBP13 NRPB12 UBP12 MTPC4 HAC1 AT2G44980 NRPC2 NRPD3B CSN2 NRPB8B CSN6A NRPD2B CSN6B CSN7 NRPB4 AT3G61800 NRPD2 AT5G28740 NRPB7 CDKD-2 NRPB8A CML20 CSN5B CSN8 AT1G19750
MMR ∩ DNA repair	1	EFS
HR ∩ DNA repair	61	RPN11 ATIM HTA2 AT3G45930 AT4G06634 UBQ8 UBQ14 AT5G05130 UBQ4 UVR8 UBQ10 HIS4 HTA6 PPX2 DSS1(I) AT4G31210 ANK1 LDL3 AT1G48360 UBQ11 PPX1 AT5G42470 HTA10 HTA5 LRS1 AT5G39770 AT1G07660 HTA3 AT1G07820 HTA12 MRG1 CDKA-1 TEB AT2G45500 MEI1 AT1G20750 UBQ13 AT5G59690 HTA13 AT1G08390 AT5G17070 AT3G07200 AT3G46320 RUB2 AT1G20720 ATM AT5G63690 RAT5 AT1G79950 RPS27AC ISE2 HEN2 AT5G59970 AT3G53730 UBC36 RPS27AB AT1G02670 RPL40B UBQ3 RPS27AA AT3G02820
NHEJ ∩ DNA repair	6	AT4G26630 AT2G32760 AT3G48710 AT5G63550 AT5G55660 AT3G03420
DNA repair	101	ATEYA UBC30 NPL41 CDC48E ckl5 AT5G25560 HMGB3 AT1G55040 AXL EMB2454 FZR2 AT1G28120 UBA1 AT5G01290 AT3G62970 CHIP AT2G25240 ADK1 AT3G16220 AT1G11800 CDC20-4 AT3G16230 CDC20-2 CHR11 AT5G18650 AT5G28210 FZR3 AT4G36080 AT4G38495 CKL13 AT3G27640 ARP6 AT1G47710 MIA40 AT1G74770 AT5G22920 AT4G24710 CKL2 AT3G45220 ckl10 ckl8 AT1G62170 UBC8 AT2G03270 AT1G27752 BSH AT1G16690 UBA2 AT2G40600 AT2G17930 UBP24 CK1 CKL6 CHR17 AT2G44510 AT1G55460 AT5G52800 UPL4 UBC29 AT2G23070 UBC9 AT5G03450 CDC48D AT5G27570 AT1G18910 AT5G53770 CDC20-1 ckl12 AT5G35970 UBC11 AT1G35530 AT5G60370 AT2G26390 AT3G01890 UEV1D CKA2 CKA1 AT2G47970 AT5G61190 AT1G34770 CDC20.3 PAPP5 UBC37 ckl7 ARP9 AT3G02200 CHR18 FY UBC10 AT2G18770 CKI1 AT1G79020 SYN4 HMGB2 TAF14 CDC48A UPL3 AT1G24290 HMGB1 FZR1 RIN1
established DNA repair	231	-not presented-

The analytical results of the gene orthology analysis are presented below. Genes shown in **bold** represent the initial genes, while genes that are not in bold represent the new genes arisen from this analysis. HGNC stands for HUGO Gene Nomenclature Committee (http://www.genenames.org) and TAIR stands for The Arabidopsis Information Resource (https://www.arabidopsis.org/index.jsp).

Table S5. DNA repair genes in human and *Arabidopsis thaliana,* grouped according to orthology.

Homo Sapiens		*Arabidopsis thaliana*		**OrthoMCL Group**
**EnsEMBL Gene**	**HGNC Symbol**	**TAIR Gene**	**Gene Name**	
ENSG00000065413<br>ENSG00000139645<br>ENSG00000206560	ANKRD44<br>ANKRD52<br>ANKRD28	**AT2G03430**<br>AT5G02620	**AT2G03430**<br>ANK1	OG5_126538
ENSG00000164508<br>ENSG00000180573<br>**ENSG00000181218**<br>ENSG00000184260<br>ENSG00000184270<br>**ENSG00000188486**<br>ENSG00000196747<br>ENSG00000196787<br>ENSG00000196866<br>ENSG00000203812<br>ENSG00000274997<br>ENSG00000276368<br>ENSG00000277075<br>ENSG00000278677	HIST1H2AA<br>HIST1H2AC<br>**HIST3H2A**<br>HIST2H2AC<br>HIST2H2AB<br>**H2AFX**<br>HIST1H2AI<br>HIST1H2AG<br>HIST1H2AD<br>HIST2H2AA3<br>HIST1H2AH<br>HIST1H2AJ<br>HIST1H2AE<br>HIST1H2AM	AT1G08880<br>AT1G51060<br>AT1G54690<br>AT3G20670<br>AT4G27230<br>AT5G02560<br>AT5G54640<br>AT5G59870	HTA5<br>HTA10<br>HTA3<br>HTA13<br>HTA2<br>HTA12<br>RAT5<br>HTA6	OG5_126570
**ENSG00000197061**<br>**ENSG00000197238**<br>**ENSG00000197837**<br>**ENSG00000278705**	**HIST1H4C**<br>**HIST1H4J**<br>**HIST4H4**<br>**HIST1H4B**	AT1G07660<br>AT1G07820<br>AT2G28740<br>AT3G45930<br>AT3G46320<br>AT3G53730<br>AT5G59690<br>AT5G59970	AT1G07660<br>AT1G07820<br>HIS4<br>AT3G45930<br>AT3G46320<br>AT3G53730<br>AT5G59690<br>AT5G59970	OG5_126573
**ENSG00000004700**<br>**ENSG00000108469**<br>**ENSG00000165392**<br>**ENSG00000197299**	**RECQL**<br>**RECQL5**<br>**WRN**<br>**BLM**	**AT1G10930**<br>**AT1G31360**<br>AT1G60930<br>AT3G05740<br>AT4G35740	**RECQL4A**<br>**MED34**<br>RECQL4B<br>RECQL1<br>RECQL3	OG5_126644
ENSG00000021355<br>ENSG00000057149<br>ENSG00000163536<br>ENSG00000206073	SERPINB1<br>SERPINB3<br>SERPINI1<br>SERPINB4	AT1G47710<br>AT1G62170<br>**AT1G64030**<br>**AT2G14540**<br>AT2G25240<br>AT2G26390<br>AT3G45220	AT1G47710<br>AT1G62170<br>**ATSRP3**<br>**ATSRP2**<br>AT2G25240<br>AT2G26390<br>AT3G45220	OG5_126693
ENSG00000013503<br>**ENSG00000047315**	POLR3B<br>**POLR2B**	AT3G18090<br>AT3G23780<br>AT4G21710<br>AT5G45140	NRPD2B<br>NRPD2<br>NRPB2<br>NRPC2	OG5_126694
**ENSG00000150991**<br>**ENSG00000170315**	**UBC**<br>**UBB**	AT1G65350<br>AT2G35635<br>AT3G09790<br>AT4G02890<br>AT4G05050<br>AT4G05320	UBQ13<br>RUB2<br>UBQ8<br>UBQ14<br>UBQ11<br>UBQ10	OG5_126703

Table S5. *Cont.*

		AT5G03240<br>AT5G20620	UBQ3<br>UBQ4	
**ENSG00000123374**<br>ENSG00000164885	**CDK2**<br>CDK5	AT3G48750	CDKA-1	OG5_126712
ENSG00000103510<br>ENSG00000136504<br>**ENSG00000172977**	KAT8<br>KAT7<br>**KAT5**	**AT5G09740**<br>**AT5G64610**	**HAM2**<br>**HAM1**	OG5_126735
**ENSG00000177302**	**TOP3A**	AT4G31210<br>**AT5G63920**	AT4G31210<br>**TOP3A**	OG5_126736
ENSG00000029993<br>**ENSG00000164104**	HMGB3<br>**HMGB2**	AT1G20693<br>AT1G20696<br>AT3G51880	HMGB2<br>HMGB3<br>HMGB1	OG5_126740
**ENSG00000105325**<br>ENSG00000117399	**FZR1**<br>CDC20	AT4G11920<br>AT4G22910<br>AT4G33260<br>AT4G33270<br>AT5G13840<br>AT5G26900<br>AT5G27080<br>AT5G27570	FZR1<br>FZR2<br>CDC20-2<br>CDC20-1<br>FZR3<br>CDC20-4<br>CDC20.3<br>AT5G27570	OG5_126765
**ENSG00000169188**	**APEX2**	**AT2G41460**<br>**AT3G48425**<br>**AT4G36050**	**ARP**<br>**APE1L**<br>**APE2**	OG5_126768
ENSG00000113712<br>**ENSG00000141551**<br>ENSG00000180138<br>**ENSG00000213923**	CSNK1A1<br>**CSNK1D**<br>CSNK1A1L<br>**CSNK1E**	AT1G03930<br>AT1G04440<br>AT1G72710<br>AT2G19470<br>AT3G23340<br>AT4G14340<br>AT4G26100<br>AT4G28540<br>AT5G43320<br>AT5G44100<br>AT5G57015	ADK1<br>CKL13<br>CKL2<br>ckl5<br>ckl10<br>CKI1<br>CK1<br>CKL6<br>ckl8<br>ckl7<br>ckl12	OG5_126780
**ENSG00000149273**	**RPS3**	**AT2G31610**<br>**AT3G53870**<br>**AT5G35530**	**RPS3A**<br>**RPS3B**<br>**RPS3C**	OG5_126820
**ENSG00000048392**<br>ENSG00000171848	**RRM2B**<br>RRM2	**AT3G23580**<br>**AT3G27060**	**RNR2A**<br>**TSO2**	OG5_126823
**ENSG00000051180**<br>**ENSG00000100206**	**RAD51**<br>**DMC1**	**AT3G22880**<br>**AT5G20850**	**DMC1**<br>**RAD51**	OG5_126834
ENSG00000072401<br>ENSG00000078967<br>**ENSG00000109332**<br>**ENSG00000131508**	UBE2D1<br>UBE2D4<br>**UBE2D3**<br>**UBE2D2**	**AT1G64230**<br>AT2G16740<br>AT3G08690<br>AT4G27960<br>AT5G41700<br>AT5G53300<br>AT5G56150	**UBC28**<br>UBC29<br>UBC11<br>UBC9<br>UBC8<br>UBC10<br>UBC30	OG5_126877

Table S5. *Cont*.

**ENSG00000116062**	**MSH6**	**AT3G24495**<br>**AT4G02070**	**MSH7**<br>**MSH6**	OG5_126895
ENSG00000070770	CSNK2A2	AT2G23070<br>**AT2G23080**<br>AT3G50000<br>AT5G67380	AT2G23070<br>**AT2G23080**<br>CKA2<br>CKA1	OG5_126901
**ENSG00000104889**	**RNASEH2A**	**AT2G25100**	**AT2G25100**	OG5_126908
**ENSG00000065057**	**NTHL1**	**AT1G05900**<br>**AT2G31450**	**NTH2**<br>**NTH1**	OG5_126913
**ENSG00000165280**	**VCP**	AT3G09840<br>AT3G53230<br>AT5G03340	CDC48A<br>CDC48D<br>CDC48E	OG5_126926
ENSG00000128951	DUT	**AT3G46940**	**DUT**	OG5_126992
**ENSG00000111445**	**RFC5**	AT1G14460<br>AT1G77470<br>AT2G02480	F14L17.24<br>RFC5<br>STI	OG5_127045
ENSG00000116030<br>ENSG00000184900<br>**ENSG00000188612**	**SUMO1**<br>SUMO3<br>**SUMO2**	AT4G26840<br>AT5G55160<br>AT5G55856	SUMO1<br>SUMO2<br>SUMO8	OG5_127053
ENSG00000133315<br>**ENSG00000138496**	MACROD1<br>**PARP9**	AT2G40600	AT2G40600	OG5_127054
ENSG00000021574<br>**ENSG00000132436**	SPAST<br>**FIGNL1**	AT2G45500<br>**AT3G27120**	AT2G45500<br>**AT3G27120**	OG5_127068
ENSG00000066117<br>ENSG00000082014<br>ENSG00000108604<br>ENSG00000108684<br>ENSG00000110881	SMARCD1<br>SMARCD3<br>SMARCD2<br>ASIC2<br>ASIC1	AT3G01890<br>**AT5G14170**	AT3G01890<br>**CHC1**	OG5_127069
ENSG00000033178<br>ENSG00000130985<br>**ENSG00000182179**	UBA6<br>UBA1<br>**UBA7**	AT2G30110<br>AT5G06460	UBA1<br>UBA2	OG5_127070
**ENSG00000085999**<br>**ENSG00000197275**	**RAD54L**<br>**RAD54B**	**AT3G19210**	**CHR25**	OG5_127098
ENSG00000102038<br>**ENSG00000153147**	SMARCA1<br>**SMARCA5**	AT3G06400<br>AT5G18620	CHR11<br>CHR17	OG5_127117
ENSG00000071794<br>ENSG00000116830	HLTF<br>TTF2	AT1G02670<br>AT5G05130<br>**AT5G22750**<br>**AT5G43530**	AT1G02670<br>AT5G05130<br>**RAD5**<br>**AT5G43530**	OG5_127144
**ENSG00000077721**<br>**ENSG00000119048**	**UBE2A**<br>**UBE2B**	**AT1G14400**<br>**AT2G02760**<br>**AT5G62540**	**UBC1**<br>**UBC2**<br>**UBC3**	OG5_127153
ENSG00000008405<br>ENSG00000121671	CRY1<br>CRY2	**AT1G04400**<br>**AT3G15620**<br>**AT4G08920**	**CRY2**<br>**UVR3**<br>**CRY1**	OG5_127186
**ENSG00000076242**	**MLH1**	**AT4G09140**	**MLH1**	OG5_127201
**ENSG00000163161**	**ERCC3**	**AT5G41360**<br>**AT5G41370**	**XPB2**<br>**XPB1**	OG5_127208
**ENSG00000198793**	**MTOR**	**AT1G50030**	**TOR**	OG5_127212

Table S5. *Cont.*

**ENSG00000143947**	**RPS27A**	AT1G23410<br>AT2G47110<br>AT3G62250	RPS27AA<br>RPS27AB<br>RPS27AC	OG5_127221
ENSG00000039123	SKIV2L2	**AT1G59760**<br>AT1G70070<br>AT2G06990	**AT1G59760**<br>ISE2<br>HEN2	OG5_127259
**ENSG00000136492**<br>**ENSG00000258366**	**BRIP1**<br>**RTEL1**	AT1G20720<br>AT1G20750<br>AT1G79950	AT1G20720<br>AT1G20750<br>AT1G79950	OG5_127294
ENSG00000187109	NAP1L1	**AT2G19480**<br>**AT4G26110**<br>**AT5G56950**	**NAP1;2**<br>**NAP1;1**<br>**NAP1;3**	OG5_127308
**ENSG00000119318**<br>**ENSG00000179262**	**RAD23B**<br>**RAD23A**	**AT1G16190**<br>**AT1G79650**<br>**AT3G02540**<br>**AT5G38470**	**RAD23A**<br>**RAD23B**<br>**RAD23C**<br>**RAD23D**	OG5_127320
**ENSG00000132646**	**PCNA**	**AT1G07370**<br>**AT2G29570**	**PCNA**<br>**PCNA2**	OG5_127352
**ENSG00000105486**	**LIG1**	**AT1G08130**<br>**AT1G49250**	**LIG1**<br>**AT1G49250**	OG5_127375
**ENSG00000149136**	**SSRP1**	**AT3G28730**	**SSRP1**	OG5_127386
**ENSG00000122008**	**POLK**	AT1G49980	AT1G49980	OG5_127393
**ENSG00000112941**<br>ENSG00000121274	**PAPD7**<br>PAPD5	AT5G53770	AT5G53770	OG5_127416
**ENSG00000076248**	**UNG**	**AT3G18630**	**ATUNG**	OG5_127417
ENSG00000163170<br>ENSG00000169627<br>ENSG00000183336	BOLA3<br>BOLA2B<br>BOLA2	**AT5G09830**	**BOLA2**	OG5_127446
**ENSG00000072501**<br>ENSG00000077935	**SMC1A**<br>SMC1B	**AT3G54670**	**TTN8**	OG5_127449
**ENSG00000035928**	**RFC1**	**AT5G22010**	**RFC1**	OG5_127465
**ENSG00000168496**	**FEN1**	**AT5G26680**	**FEN1**	OG5_127472
ENSG00000105011<br>**ENSG00000111875**	ASF1B<br>**ASF1A**	**AT1G66740**<br>**AT5G38110**	**ASF1A**<br>**ASF1B**	OG5_127499
**ENSG00000174371**	**EXO1**	**AT1G18090**<br>AT1G29630	**AT1G18090**<br>EXO1	OG5_127511
**ENSG00000095002**	**MSH2**	**AT3G18524**	**MSH2**	OG5_127538
**ENSG00000132383**	**RPA1**	**AT2G06510**<br>AT4G19130<br>**AT5G08020**<br>**AT5G45400**<br>**AT5G61000**	**RPA1A**<br>RPA1E<br>**RPA1B**<br>**RPA1C**<br>**RPA1D**	OG5_127539
**ENSG00000104884**	**ERCC2**	**AT1G03190**	**UVH6**	OG5_127585
**ENSG00000163312**	**HELQ**	AT4G32700	TEB	OG5_127591
**ENSG00000049541**	**RFC2**	AT1G63160	RFC2	OG5_127600
**ENSG00000102978**	**POLR2C**	AT2G15400<br>AT2G15430	NRPD3B<br>NRPB3	OG5_127622
**ENSG00000149923**	**PPP4C**	AT4G26720<br>AT5G55260	PPX1<br>PPX2	OG5_127639

Table S5. *Cont.*

**ENSG00000177700**	**POLR2L**	AT1G11475<br>AT1G61700	NRPB10<br>NRPB10L	OG5_127691
**ENSG00000177889**	**UBE2N**	AT1G16890<br>**AT1G78870**	UBC36<br>**UBC35**	OG5_127739
ENSG00000163029	SMC6	**AT5G07660**<br>**AT5G61460**	**SMC6A**<br>**SMC6B**	OG5_127751
**ENSG00000115233**	**PSMD14**	AT5G23540	RPN11	OG5_127757
**ENSG00000133119**	**RFC3**	AT5G27740	RFC3	OG5_127765
ENSG00000124208<br>**ENSG00000244687**	TMEM189<br>-UBE2V1<br>**UBE2V1**	**AT1G23260**<br>**AT1G70660**<br>**AT2G36060**<br>AT3G52560	**UEV1A**<br>**UEV1B**<br>**UEV1C**<br>UEV1D	OG5_127771
**ENSG00000187555**	**USP7**	AT3G11910<br>AT5G06600	UBP13<br>UBP12	OG5_127773
**ENSG00000108055**	**SMC3**	**AT2G27170**	**SMC3**	OG5_127789
**ENSG00000145736**<br>**ENSG00000183474**	**GTF2H2**<br>**GTF2H2C**	**AT1G05055**	**ATGTF2H2**	OG5_127799
**ENSG00000106628**	**POLD2**	AT2G42120	POLD2	OG5_127874
ENSG00000121211	MND1	**AT4G29170**	**MND1**	OG5_127882
**ENSG00000163882**	**POLR2H**	AT1G54250<br>AT3G59600	NRPB8A<br>NRPB8B	OG5_127936
**ENSG00000175792**	**RUVBL1**	AT5G22330	RIN1	OG5_127944
**ENSG00000020922**	**MRE11A**	**AT5G54260**	**MRE11**	OG5_127969
**ENSG00000175595**	**ERCC4**	**AT5G41150**	**UVH1**	OG5_127977
**ENSG00000168002**	**POLR2G**	AT3G22900<br>AT5G59180	NRPD7<br>NRPB7	OG5_127981
**ENSG00000100387**	**RBX1**	AT3G42830<br>AT5G20570	RBX1B<br>RBX1A	OG5_127986
**ENSG00000114026**	**OGG1**	**AT1G21710**	**OGG1**	OG5_127994
**ENSG00000096401**	**CDC5L**	**AT1G09770**	**CDC5**	OG5_128000
**ENSG00000122512**	**PMS2**	**AT4G02460**	**PMS1**	OG5_128001
ENSG00000111880	RNGTT	**AT3G09100**<br>AT5G01290<br>AT5G28210	**AT3G09100**<br>AT5G01290<br>AT5G28210	OG5_128005
**ENSG00000163918**	**RFC4**	AT1G21690	RFC4	OG5_128040
**ENSG00000136709**	**WDR33**	AT5G13480	FY	OG5_128052
**ENSG00000092201**	**SUPT16H**	**AT4G10710**	**SPT16**	OG5_128055
**ENSG00000124535**	**WRNIP1**	AT1G24290	AT1G24290	OG5_128067
**ENSG00000132781**	**MUTYH**	**AT4G12740**	**MYH**	OG5_128159
**ENSG00000110107**	**PRPF19**	**AT1G04510**<br>AT2G33340	**PRP19A**<br>PRP19B	OG5_128187
**ENSG00000105258**	**POLR2I**	**AT3G16980**<br>**AT4G16265**	**NRPB9A**<br>**NRPB9B**	OG5_128243
**ENSG00000011485**	**PPP5C**	AT2G42810	PAPP5	OG5_128254
**ENSG00000221983**	**UBA52**	AT2G36170<br>AT3G52590	RPL40B<br>RPL40B	OG5_128257
**ENSG00000100479**	**POLE2**	**AT5G22110**	**ATDPB2**	OG5_128285
**ENSG00000121988**<br>ENSG00000138375	**ZRANB3**<br>SMARCAL1	AT1G48310	CHR18	OG5_128295

Table S5. *Cont.*

ENSG00000077080<br>**ENSG00000136518**	ACTL6B<br>**ACTL6A**	**AT1G18450**	**ARP4**	OG5_128301
**ENSG00000039650**	**PNKP**	**AT3G14890**	**ZDP**	OG5_128311
**ENSG00000128731**	**HERC2**	AT5G63860	UVR8	OG5_128314
**ENSG00000163743**	**RCHY1**	AT1G18910<br>AT1G74770<br>AT3G18290<br>AT3G62970<br>AT5G18650<br>AT5G22920<br>AT5G25560	AT1G18910<br>AT1G74770<br>EMB2454<br>AT3G62970<br>AT5G18650<br>AT5G22920<br>AT5G25560	OG5_128327
**ENSG00000240682**<br>ENSG00000261796	**ISY1**<br>ISY1-RAB43	AT3G18790	AT3G18790	OG5_128335
ENSG00000144867	SRPRB	AT2G18770<br>**AT5G05670**	AT2G18770<br>**AT5G05670**	OG5_128347
**ENSG00000076924**	**XAB2**	AT5G28740	AT5G28740	OG5_128360
ENSG00000060762	MPC1	**AT5G20090**	**MPC1**	OG5_128371
**ENSG00000175054**	**ATR**	**AT5G40820**	**ATR**	OG5_128386
**ENSG00000159259**	**CHAF1B**	**AT5G64630**	**FAS2**	OG5_128392
ENSG00000159593	NAE1	**AT1G05180**<br>AT2G32410	**AXR1**<br>AXL	OG5_128412
**ENSG00000129484**<br>**ENSG00000143799**	**PARP2**<br>**PARP1**	**AT2G31320**<br>**AT4G02390**	**PARP1**<br>**PARP2**	OG5_128423
**ENSG00000121022**	**COPS5**	AT1G22920<br>AT1G71230	CSN5B<br>CSN5A	OG5_128438
**ENSG00000153827**	**TRIP12**	AT4G38600<br>AT5G02880	UPL3<br>UPL4	OG5_128439
**ENSG00000132740**	**IGHMBP2**	AT2G03270<br>AT5G35970	AT2G03270<br>AT5G35970	OG5_128468
**ENSG00000131153**	**GINS2**	**AT3G12530**	**GINS2**	OG5_128484
ENSG00000127337	YEATS4	AT2G18000<br>**AT5G45600**	TAF14<br>**TAF14B**	OG5_128501
**ENSG00000170734**	**POLH**	**AT5G44740**	**POLH**	OG5_128521
**ENSG00000154767**	**XPC**	**AT5G16630**	**ATRAD4**	OG5_128561
**ENSG00000182446**	**NPLOC4**	AT2G47970<br>AT3G63000	AT2G47970<br>NPL41	OG5_128565
ENSG00000120616<br>**ENSG00000135999**	EPC1<br>**EPC2**	AT1G16690<br>AT1G79020	AT1G16690<br>AT1G79020	OG5_128576
**ENSG00000151657**	**KIN**	AT1G55460	AT1G55460	OG5_128587
**ENSG00000144231**	**POLR2D**	AT5G09920	NRPB4	OG5_128606
**ENSG00000198887**	**SMC5**	**AT5G15920**	**SMC5**	OG5_128615
**ENSG00000135945**	**REV1**	**AT5G44750**	**REV1**	OG5_128628
**ENSG00000187790**	**FANCM**	AT1G35530	AT1G35530	OG5_128649
**ENSG00000009413**	**REV3L**	AT1G55040<br>**AT1G67500**	AT1G55040<br>**REV3**	OG5_128651
ENSG00000176635	HORMAD2	**AT1G67370**	**ASY1**	OG5_128667
**ENSG00000134899**	**ERCC5**	**AT3G28030**	**UVH3**	OG5_128675
**ENSG00000166200**	**COPS2**	AT2G26990	CSN2	OG5_128688
**ENSG00000012061**	**ERCC1**	**AT3G05210**	**ERCC1**	OG5_128697

Table S5. *Cont.*

**ENSG00000132207**<br>**ENSG00000181625**	**SLX1A**<br>**SLX1B**	**AT2G30350**	**AT2G30350**	OG5_128732
**ENSG00000225830**	**ERCC6**	**AT2G18760**	**CHR8**	OG5_128748
**ENSG00000227345**	**PARG**	AT2G31865<br>AT2G31870	PARG2<br>PARG1	OG5_128766
**ENSG00000117748**	**RPA2**	**AT2G24490**<br>**AT3G02920**	**RPA2A**<br>**RPA2B**	OG5_128774
ENSG00000014138	POLA2	**AT1G67630**	**POLA2**	OG5_128793
**ENSG00000181555**	**SETD2**	AT1G77300	EFS	OG5_128830
**ENSG00000147400**<br>ENSG00000177143	**CETN2**<br>CETN1	AT3G50360	CML20	OG5_128884
**ENSG00000213780**<br>**ENSG00000221974**<br>**ENSG00000226384**<br>**ENSG00000233149**<br>**ENSG00000234370**<br>**ENSG00000236895**	**GTF2H4**<br>**GTF2H4**<br>**GTF2H4**<br>**GTF2H4**<br>**GTF2H4**<br>**GTF2H4**	**AT4G17020**	**AT4G17020**	OG5_128887
**ENSG00000147536**	**GINS4**	**AT5G49010**	**SLD5**	OG5_128888
**ENSG00000196367**	**TRRAP**	AT2G17930<br>AT4G36080	AT2G17930<br>AT4G36080	OG5_128900
**ENSG00000185787**	**MORF4L1**	AT4G37280	MRG1	OG5_128923
**ENSG00000139842**<br>**ENSG00000158290**	**CUL4A**<br>**CUL4B**	**AT5G46210**	**CUL4**	OG5_128927
**ENSG00000163781**	**TOPBP1**	AT1G77320	MEI1	OG5_128953
**ENSG00000149311**	**ATM**	AT3G48190	ATM	OG5_128955
**ENSG00000166896**	**XRCC6BP1**	AT3G03420	AT3G03420	OG5_128965
ENSG00000089723<br>**ENSG00000167770**	OTUB2<br>**OTUB1**	AT1G28120	AT1G28120	OG5_128982
**ENSG00000178028**	**DMAP1**	**AT2G47210**	**SWC4**	OG5_128988
**ENSG00000071539**	**TRIP13**	AT4G24710	AT4G24710	OG5_128995
**ENSG00000107949**	**BCCIP**	AT2G44510	AT2G44510	OG5_129004
**ENSG00000111652**<br>ENSG00000144524	**COPS7A**<br>COPS7B	AT1G02090	CSN7	OG5_129008
**ENSG00000125885**	**MCM8**	**AT3G09660**	**MCM8**	OG5_129017
**ENSG00000042088**	**TDP1**	**AT5G15170**	**TDP1**	OG5_129037
**ENSG00000107672**	**NSMCE4A**	**AT1G51130**<br>**AT3G20760**	**NSE4A**<br>**NSE4B**	OG5_129062
**ENSG00000196419**	**XRCC6**	**AT1G16970**	**KU70**	OG5_129086
**ENSG00000198924**	**DCLRE1A**	AT2G45700<br>**AT3G26680**	AT2G45700<br>**SNM1**	OG5_129094
**ENSG00000111358**	**GTF2H3**	**AT1G18340**	**AT1G18340**	OG5_129096
ENSG00000141446<br>**ENSG00000171320**	ESCO1<br>**ESCO2**	**AT4G31400**	**CTF7**	OG5_129141
**ENSG00000172732**	**MUS81**	**AT4G30870**<br>AT5G39770	**MUS81**<br>AT5G39770	OG5_129162
ENSG00000005339<br>**ENSG00000100393**	CREBBP<br>**EP300**	AT1G16710<br>AT1G55970<br>AT1G67220<br>AT1G79000<br>AT3G12980	HAC12<br>HAC04<br>HAC2<br>HAC1<br>HAC5	OG5_129174

Table S5. *Cont.*

**ENSG00000134058**	**CDK7**	AT1G18040<br>AT1G66750<br>AT1G73690	CDKD-3<br>CDKD-2<br>CDKD-1	OG5_129224
ENSG00000149100	EIF3M	AT3G02200<br>**AT5G15610**	AT3G02200<br>**AT5G15610**	OG5_129237
**ENSG00000134480**	**CCNH**	AT5G27620	CYCH1-1	OG5_129254
**ENSG00000103194**	**USP10**	AT4G30890	UBP24	OG5_129274
**ENSG00000163104**	**SMARCAD1**	**AT2G02090**	**ETL1**	OG5_129286
**ENSG00000093009**	**CDC45**	**AT3G25100**	**CDC45**	OG5_129331
**ENSG00000137074**	**APTX**	**AT5G01310**	**BHLH140**	OG5_129343
**ENSG00000079246**	**XRCC5**	**AT1G48050**	**KU80**	OG5_129372
**ENSG00000227314**<br>**ENSG00000230293**<br>**ENSG00000235222**<br>**ENSG00000235569**	**MSH5**<br>**MSH5**<br>**MSH5**<br>**MSH5**	**AT3G20475**	**MSH5**	OG5_129379
**ENSG00000152942**	**RAD17**	**AT5G66130**	**RAD17**	OG5_129387
**ENSG00000021776**	**AQR**	AT2G38770	EMB2765	OG5_129398
**ENSG00000085224**	**ATRX**	**AT1G08600**	**ATRX**	OG5_129416
ENSG00000134453	RBM17	**AT1G30480**	**DRT111**	OG5_129437
ENSG00000138678<br>ENSG00000158669	GPAT3<br>GPAT4	**AT5G60620**	**GPAT9**	OG5_129480
**ENSG00000164754**	**RAD21**	AT5G16270	SYN4	OG5_129513
**ENSG00000100601**	**ALKBH1**	**AT1G11780**	**AT1G11780**	OG5_129589
ENSG00000151092	NGLY1	**AT5G49570**	**PNG1**	OG5_129623
**ENSG00000138346**	**DNA2**	AT1G08840	emb2411	OG5_129631
**ENSG00000163528**	**CHCHD4**	AT5G23395	MIA40	OG5_129637
**ENSG00000138663**	**COPS4**	AT5G42970	CSN4	OG5_129642
**ENSG00000167986**	**DDB1**	**AT4G05420**<br>**AT4G21100**	**DDB1A**<br>**DDB1B**	OG5_129644
**ENSG00000169189**	**NSMCE1**	**AT5G21140**	**emb1379**	OG5_129651
**ENSG00000075089**	**ACTR6**	AT3G33520	ARP6	OG5_129661
**ENSG00000104313**<br>**ENSG00000112319**	**EYA1**<br>**EYA4**	AT2G35320	ATEYA	OG5_129664
**ENSG00000141030**	**COPS3**	AT5G14250	CSN3	OG5_129714
**ENSG00000114742**	**WDR48**	AT3G05090	LRS1	OG5_129752
**ENSG00000167670**	**CHAF1A**	**AT1G65470**	**FAS1**	OG5_129755
**ENSG00000068878**	**PSME4**	**AT3G13330**	**PA200**	OG5_129781
**ENSG00000103266**	**STUB1**	AT3G07370	CHIP	OG5_129825
**ENSG00000099956**	**SMARCB1**	AT3G17590	BSH	OG5_129866
**ENSG00000151164**<br>**ENSG00000172613**	**RAD9B**<br>**RAD9A**	**AT3G05480**	**RAD9**	OG5_129960
**ENSG00000110768**	**GTF2H1**	**AT1G55750**<br>**AT3G61420**	**TFB1-1**<br>**TFB1-3**	OG5_129968
ENSG00000273899	NOL12	**AT1G11240**	**AT1G11240**	OG5_129994
**ENSG00000111877**	**MCM9**	AT2G14050	MCM9	OG5_130040
**ENSG00000057468**	**MSH4**	**AT4G17380**	**MSH4**	OG5_130077
**ENSG00000077463**	**SIRT6**	AT5G55760	SRT1	OG5_130121
**ENSG00000147669**	**POLR2K**	AT5G41010	NRPB12	OG5_130124
**ENSG00000174405**	**LIG4**	**AT5G57160**	**LIG4**	OG5_130132
**ENSG00000103152**	**MPG**	**AT3G12040**	**MAG**	OG5_130143

Table S5. *Cont.*

**ENSG00000127922**	**SHFM1**	AT1G64750<br>**AT5G45010**	DSS1(I)<br>**ATDSS1(V)**	OG5_130158
ENSG00000197771	MCMBP	**AT2G40550**	**ETG1**	OG5_130164
**ENSG00000168090**	**COPS6**	AT4G26430<br>AT5G56280	CSN6B<br>CSN6A	OG5_130197
**ENSG00000153391**	**INO80C**	AT4G38495	AT4G38495	OG5_130230
**ENSG00000101442**	**ACTR5**	**AT3G12380**	**ARP5**	OG5_130234
**ENSG00000075131**	**TIPIN**	AT3G02820	AT3G02820	OG5_130239
**ENSG00000128908**	**INO80**	**AT3G57300**	**INO80**	OG5_130269
**ENSG00000113318**	**MSH3**	**AT4G25540**	**MSH3**	OG5_130351
**ENSG00000198690**	**FAN1**	AT1G48360	AT1G48360	OG5_130379
**ENSG00000113456**	**RAD1**	**AT4G17760**	**AT4G17760**	OG5_130438
**ENSG00000004487**	**KDM1A**	AT4G16310	LDL3	OG5_130448
**ENSG00000113812**	**ACTR8**	AT5G43500	ARP9	OG5_130536
**ENSG00000119684**	**MLH3**	**AT4G35520**	**MLH3**	OG5_130552
**ENSG00000077514**	**POLD3**	**AT1G78650**	**POLD3**	OG5_130668
**ENSG00000143476**	**DTL**	AT3G27640	AT3G27640	OG5_130781
**ENSG00000124795**	**DEK**	AT3G48710<br>AT4G26630<br>AT5G55660<br>AT5G63550	AT3G48710<br>AT4G26630<br>AT5G55660<br>AT5G63550	OG5_130851
**ENSG00000138303**	**ASCC1**	AT3G16220<br>AT3G16230	AT3G16220<br>AT3G16230	OG5_130866
**ENSG00000049167**	**ERCC8**	AT1G19750<br>AT1G27840	AT1G19750<br>ATCSA-1	OG5_130892
**ENSG00000139579**<br>**ENSG00000173559**	**NABP2**<br>**NABP1**	AT5G63690	AT5G63690	OG5_130959
**ENSG00000126215**	**XRCC3**	**AT5G57450**	**XRCC3**	OG5_131019
**ENSG00000108384**	**RAD51C**	**AT2G45280**	**RAD51C**	OG5_131246
ENSG00000185838	GNB1L	**AT4G29860**	**DSE1**	OG5_131248
**ENSG00000156831**	**NSMCE2**	**AT3G15150**	**MMS21**	OG5_131427
**ENSG00000014824**	**SLC30A9**	AT1G51610	MTPC4	OG5_131446
**ENSG00000116670**	**MAD2L2**	**AT1G16590**	**REV7**	OG5_131503
**ENSG00000168411**	**RFWD3**	AT5G03450	AT5G03450	OG5_131611
**ENSG00000164002**	**EXO5**	AT5G60370	AT5G60370	OG5_131645
**ENSG00000198382**	**UVRAG**	AT2G32760	AT2G32760	OG5_131718
**ENSG00000198783**	**ZNF830**	AT3G02860	AT3G02860	OG5_131737
**ENSG00000111802**	**TDP2**	AT1G11800	AT1G11800	OG5_131766
**ENSG00000111602**	**TIMELESS**	AT5G52910	ATIM	OG5_131860
**ENSG00000139618**	**BRCA2**	AT4G00020<br>**AT5G01630**	BRCA2A<br>**BRCA2B**	OG5_131863
**ENSG00000164306**	**PRIMPOL**	AT5G52800	AT5G52800	OG5_131904
**ENSG00000140525**	**FANCI**	**AT5G49110**	**AT5G49110**	OG5_131962
**ENSG00000166169**	**POLL**	**AT1G10520**	**AT1G10520**	OG5_132021
**ENSG00000198612**	**COPS8**	AT4G14110	CSN8	OG5_132111
ENSG00000143207	RFWD2	**AT2G32950**	**COP1**	OG5_132181
**ENSG00000163605**	**PPP4R2**	AT5G17070	AT5G17070	OG5_132213
**ENSG00000115392**	**FANCL**	**AT5G65740**	**AT5G65740**	OG5_132368
ENSG00000140543	DET1	**AT4G10180**	**DET1**	OG5_132401
ENSG00000167740	CYB5D2	**AT4G14965**	**MAPR4**	OG5_132425

Table S5. *Cont.*

**ENSG00000100325**	**ASCC2**	AT1G27752	AT1G27752	OG5_132432
**ENSG00000182150**	**ERCC6L2**	**AT1G03750**	**SWI2**	OG5_132588
**ENSG00000175482**	**POLD4**	AT1G09815	POLD4	OG5_132598
**ENSG00000144554**	**FANCD2**	**AT4G14970**	**AT4G14970**	OG5_132711
**ENSG00000073050**	**XRCC1**	**AT1G80420**	**XRCC1**	OG5_132817
**ENSG00000100811**<br>ENSG00000230797	**YY1**<br>YY2	AT4G06634	AT4G06634	OG5_132824
**ENSG00000185379**	**RAD51D**	AT1G07745	RAD51D	OG5_132909
ENSG00000152253	SPC25	**AT3G48210**	**AT3G48210**	OG5_132949
**ENSG00000104320**	**NBN**	**AT3G02680**	**NBS1**	OG5_133106
**ENSG00000077152**	**UBE2T**	AT3G24515	UBC37	OG5_133146
**ENSG00000196584**	**XRCC2**	**AT5G64520**	**XRCC2**	OG5_133234
**ENSG00000079616**	**KIF22**	**AT5G02370**	**AT5G02370**	OG5_133348
**ENSG00000214941**	**ZSWIM7**	**AT4G33925**	**AT4G33925**	OG5_133488
**ENSG00000163945**	**UVSSA**	AT3G61800	AT3G61800	OG5_133565
**ENSG00000185115**	**NDNL2**	AT1G34770	AT1G34770	OG5_133962
**ENSG00000178295**	**GEN1**	**AT1G01880**	**GEN1**	OG5_134343
**ENSG00000166783**	**KIAA0430**	AT5G61190	AT5G61190	OG5_134363
**ENSG00000198890**	**PRMT6**	AT3G20020	PRMT6	OG5_134380
**ENSG00000166199**	**ALKBH3**	**AT2G22260**	**ALKBH2**	OG5_134523
**ENSG00000063978**	**RNF4**	AT3G07200	AT3G07200	OG5_134631
**ENSG00000118655**	**DCLRE1B**	AT1G27410	AT1G27420/<br>F17L21.21	OG5_135047
**ENSG00000131778**	**CHD1L**	AT2G44980	AT2G44980	OG5_135107
**ENSG00000152422**	**XRCC4**	**AT3G23100**	**XRCC4**	OG5_135131
**ENSG00000182185**	**RAD51B**	**AT2G28560**	**RAD51B**	OG5_135350
**ENSG00000158019**	**BRE**	AT5G42470	AT5G42470	OG5_135444
**ENSG00000134574**	**DDB2**	**AT5G58760**	**DDB2**	OG5_136585
**ENSG00000175643**	**RMI2**	AT1G08390	AT1G08390	OG5_139541
**ENSG00000154328**	**NEIL2**	**AT1G52500**	**FPG1**	OG5_147326

Table S6. BER genes in human and *Arabidopsis thaliana,* grouped according to orthology.

Homo sapiens		*Arabidopsis thaliana*		**OrthoMCL Group**
**EnsEMBL Gene**	**HGNC Symbol**	**TAIR Gene**	**Gene Name**	
ENSG00000004700<br>ENSG00000108469<br>**ENSG00000165392**<br>ENSG00000197299	RECQL<br>RECQL5<br>**WRN**<br>BLM	AT1G10930<br>AT1G31360<br>AT1G60930<br>AT3G05740<br>AT4G35740	RECQL4A<br>MED34<br>RECQL4B<br>RECQL1<br>RECQL3	OG5_126644
**ENSG00000169188**	**APEX2**	**AT2G41460**<br>**AT3G48425**<br>**AT4G36050**	**ARP**<br>**APE1L**<br>**APE2**	OG5_126768
**ENSG00000065057**	**NTHL1**	**AT1G05900**<br>**AT2G31450**	**NTH2**<br>**NTH1**	OG5_126913
**ENSG00000111445**	**RFC5**	AT1G14460<br>AT1G77470<br>AT2G02480	F14L17.24<br>RFC5<br>STI	OG5_127045

Table S6. *Cont.*

**ENSG00000132646**	**PCNA**	AT1G07370<br>AT2G29570	PCNA<br>PCNA2	OG5_127352
**ENSG00000105486**	**LIG1**	AT1G08130<br>AT1G49250	LIG1<br>AT1G49250	OG5_127375
**ENSG00000076248**	**UNG**	**AT3G18630**	**ATUNG**	OG5_127417
**ENSG00000035928**	**RFC1**	AT5G22010	RFC1	OG5_127465
**ENSG00000168496**	**FEN1**	**AT5G26680**	**FEN1**	OG5_127472
**ENSG00000132383**	**RPA1**	AT2G06510<br>AT4G19130<br>AT5G08020<br>AT5G45400<br>AT5G61000	RPA1A<br>RPA1E<br>RPA1B<br>RPA1C<br>RPA1D	OG5_127539
**ENSG00000049541**	**RFC2**	AT1G63160	RFC2	OG5_127600
**ENSG00000133119**	**RFC3**	AT5G27740	RFC3	OG5_127765
**ENSG00000106628**	**POLD2**	AT2G42120	POLD2	OG5_127874
**ENSG00000020922**	**MRE11A**	**AT5G54260**	**MRE11**	OG5_127969
**ENSG00000114026**	**OGG1**	**AT1G21710**	**OGG1**	OG5_127994
**ENSG00000163918**	**RFC4**	AT1G21690	RFC4	OG5_128040
**ENSG00000132781**	**MUTYH**	**AT4G12740**	**MYH**	OG5_128159
**ENSG00000039650**	**PNKP**	AT3G14890	ZDP	OG5_128311
**ENSG00000129484**<br>**ENSG00000143799**	**PARP2**<br>**PARP1**	AT2G31320<br>AT4G02390	PARP1<br>PARP2	OG5_128423
**ENSG00000225830**	**ERCC6**	AT2G18760	CHR8	OG5_128748
**ENSG00000227345**	**PARG**	AT2G31865<br>AT2G31870	PARG2<br>PARG1	OG5_128766
**ENSG00000117748**	**RPA2**	AT2G24490<br>AT3G02920	RPA2A<br>RPA2B	OG5_128774
**ENSG00000138346**	**DNA2**	AT1G08840	emb2411	OG5_129631
**ENSG00000077463**	**SIRT6**	AT5G55760	SRT1	OG5_130121
**ENSG00000103152**	**MPG**	**AT3G12040**	**MAG**	OG5_130143
**ENSG00000077514**	**POLD3**	AT1G78650	POLD3	OG5_130668
**ENSG00000175482**	**POLD4**	AT1G09815	POLD4	OG5_132598
**ENSG00000073050**	**XRCC1**	AT1G80420	XRCC1	OG5_132817
**ENSG00000198890**	**PRMT6**	AT3G20020	PRMT6	OG5_134380
**ENSG00000154328**	**NEIL2**	**AT1G52500**	**FPG1**	OG5_147326

Table S7. NER genes in human and *Arabidopsis thaliana*, grouped according to orthology.

Homo sapiens		***Arabidopsis thaliana***		**OrthoMCL Group**
**EnsEMBL Gene**	**HGNC Symbol**	**TAIR Gene**	**Gene Name**	
ENSG00000013503<br>**ENSG00000047315**	POLR3B<br>**POLR2B**	AT3G18090<br>AT3G23780<br>AT4G21710<br>AT5G45140	NRPD2B<br>NRPD2<br>NRPB2<br>NRPC2	OG5_126694
**ENSG00000116030**<br>**ENSG00000184900**<br>**ENSG00000188612**	**SUMO1**<br>**SUMO3**<br>**SUMO2**	AT4G26840<br>AT5G55160<br>AT5G55856	SUMO1<br>SUMO2<br>SUMO8	OG5_127053
**ENSG00000163161**	ERCC3	**AT5G41360**<br>**AT5G41370**	**XPB2**<br>**XPB1**	OG5_127208

Table S7. *Cont.*

**ENSG00000119318**<br>**ENSG00000179262**	**RAD23B**<br>**RAD23A**	**AT1G16190**<br>**AT1G79650**<br>**AT3G02540**<br>**AT5G38470**	**RAD23A**<br>**RAD23B**<br>**RAD23C**<br>**RAD23D**	OG5_127320
**ENSG00000122008**	**POLK**	AT1G49980	AT1G49980	OG5_127393
**ENSG00000104884**	**ERCC2**	**AT1G03190**	**UVH6**	OG5_127585
**ENSG00000102978**	**POLR2C**	AT2G15400<br>AT2G15430	NRPD3B<br>NRPB3	OG5_127622
**ENSG00000177700**	**POLR2L**	AT1G11475<br>AT1G61700	NRPB10<br>NRPB10L	OG5_127691
**ENSG00000187555**	**USP7**	AT3G11910<br>AT5G06600	UBP13<br>UBP12	OG5_127773
**ENSG00000145736**<br>**ENSG00000183474**	**GTF2H2**<br>**GTF2H2C**	**AT1G05055**	**ATGTF2H2**	OG5_127799
**ENSG00000163882**	**POLR2H**	AT1G54250<br>AT3G59600	NRPB8A<br>NRPB8B	OG5_127936
**ENSG00000168002**	**POLR2G**	AT3G22900<br>AT5G59180	NRPD7<br>NRPB7	OG5_127981
**ENSG00000100387**	**RBX1**	AT3G42830<br>AT5G20570	RBX1B<br>RBX1A	OG5_127986
**ENSG00000110107**	**PRPF19**	AT1G04510<br>AT2G33340	PRP19A<br>PRP19B	OG5_128187
**ENSG00000105258**	**POLR2I**	**AT3G16980**<br>**AT4G16265**	**NRPB9A**<br>**NRPB9B**	OG5_128243
**ENSG00000240682**<br>ENSG00000261796	**ISY1**<br>ISY1-RAB43	AT3G18790	AT3G18790	OG5_128335
**ENSG00000076924**	**XAB2**	AT5G28740	AT5G28740	OG5_128360
**ENSG00000121022**	**COPS5**	AT1G22920<br>AT1G71230	CSN5B<br>CSN5A	OG5_128438
**ENSG00000154767**	**XPC**	**AT5G16630**	**ATRAD4**	OG5_128561
**ENSG00000144231**	**POLR2D**	AT5G09920	NRPB4	OG5_128606
**ENSG00000134899**	**ERCC5**	**AT3G28030**	**UVH3**	OG5_128675
**ENSG00000166200**	**COPS2**	AT2G26990	CSN2	OG5_128688
**ENSG00000147400**<br>ENSG00000177143	**CETN2**<br>CETN1	AT3G50360	CML20	OG5_128884
**ENSG00000213780**<br>**ENSG00000221974**<br>**ENSG00000226384**<br>**ENSG00000233149**<br>**ENSG00000234370**<br>**ENSG00000236895**	**GTF2H4**<br>**GTF2H4**<br>**GTF2H4**<br>**GTF2H4**<br>**GTF2H4**<br>**GTF2H4**	**AT4G17020**	**AT4G17020**	OG5_128887
**ENSG00000139842**<br>**ENSG00000158290**	**CUL4A**<br>**CUL4B**	AT5G46210	CUL4	OG5_128927
**ENSG00000111652**<br>**ENSG00000144524**	**COPS7A**<br>**COPS7B**	AT1G02090	CSN7	OG5_129008
**ENSG00000198924**	**DCLRE1A**	AT2G45700<br>AT3G26680	AT2G45700<br>SNM1	OG5_129094
**ENSG00000111358**	**GTF2H3**	**AT1G18340**	**AT1G18340**	OG5_129096

Table S7. *Cont.*

ENSG00000005339<br>**ENSG00000100393**	CREBBP<br>**EP300**	AT1G16710<br>AT1G55970<br>AT1G67220<br>AT1G79000<br>AT3G12980	HAC12<br>HAC04<br>HAC2<br>HAC1<br>HAC5	OG5_129174
**ENSG00000134058**	**CDK7**	AT1G18040<br>AT1G66750<br>AT1G73690	CDKD-3<br>CDKD-2<br>CDKD-1	OG5_129224
**ENSG00000134480**	**CCNH**	AT5G27620	CYCH1-1	OG5_129254
**ENSG00000021776**	**AQR**	AT2G38770	EMB2765	OG5_129398
ENSG00000151092	NGLY1	**AT5G49570**	**PNG1**	OG5_129623
**ENSG00000138663**	**COPS4**	AT5G42970	CSN4	OG5_129642
**ENSG00000167986**	**DDB1**	**AT4G05420**<br>**AT4G21100**	**DDB1A**<br>**DDB1B**	OG5_129644
**ENSG00000141030**	**COPS3**	AT5G14250	CSN3	OG5_129714
**ENSG00000110768**	**GTF2H1**	**AT1G55750**<br>**AT3G61420**	**TFB1-1**<br>**TFB1-3**	OG5_129968
**ENSG00000147669**	**POLR2K**	AT5G41010	NRPB12	OG5_130124
**ENSG00000174405**	**LIG4**	**AT5G57160**	**LIG4**	OG5_130132
**ENSG00000168090**	**COPS6**	AT4G26430<br>AT5G56280	CSN6B<br>CSN6A	OG5_130197
**ENSG00000049167**	**ERCC8**	AT1G19750<br>AT1G27840	AT1G19750<br>ATCSA-1	OG5_130892
**ENSG00000014824**	**SLC30A9**	AT1G51610	MTPC4	OG5_131446
**ENSG00000198783**	**ZNF830**	AT3G02860	AT3G02860	OG5_131737
**ENSG00000166169**	**POLL**	**AT1G10520**	**AT1G10520**	OG5_132021
**ENSG00000198612**	**COPS8**	AT4G14110	CSN8	OG5_132111
**ENSG00000163945**	**UVSSA**	AT3G61800	AT3G61800	OG5_133565
**ENSG00000118655**	**DCLRE1B**	AT1G27410	AT1G27420/<br>F17L21.21	OG5_135047
**ENSG00000131778**	**CHD1L**	AT2G44980	AT2G44980	OG5_135107
**ENSG00000134574**	**DDB2**	AT5G58760	DDB2	OG5_136585

Table S8. MMR genes in human and *Arabidopsis thaliana*, grouped according to orthology.

Homo sapiens		*Arabidopsis thaliana*		**OrthoMCL Group**
**EnsEMBL Gene**	**HGNC Symbol**	**TAIR Gene**	**Gene Name**	
**ENSG00000116062**	**MSH6**	**AT3G24495**<br>**AT4G02070**	**MSH7**<br>**MSH6**	OG5_126895
**ENSG00000104889**	**RNASEH2A**	**AT2G25100**	**AT2G25100**	OG5_126908
**ENSG00000076242**	**MLH1**	**AT4G09140**	**MLH1**	OG5_127201
**ENSG00000132646**	**PCNA**	**AT1G07370**<br>**AT2G29570**	**PCNA**<br>**PCNA2**	OG5_127352
**ENSG00000095002**	**MSH2**	**AT3G18524**	**MSH2**	OG5_127538
**ENSG00000122512**	**PMS2**	**AT4G02460**	**PMS1**	OG5_128001
**ENSG00000181555**	**SETD2**	AT1G77300	EFS	OG5_128830
**ENSG00000227314**<br>**ENSG00000230293**<br>**ENSG00000235222**<br>**ENSG00000235569**	**MSH5**<br>**MSH5**<br>**MSH5**<br>**MSH5**	**AT3G20475**	**MSH5**	OG5_129379
**ENSG00000057468**	**MSH4**	**AT4G17380**	**MSH4**	OG5_130077
**ENSG00000113318**	**MSH3**	**AT4G25540**	**MSH3**	OG5_130351
**ENSG00000119684**	**MLH3**	**AT4G35520**	**MLH3**	OG5_130552

Table S9. HR genes in human and *Arabidopsis thaliana,* grouped according to orthology.

Homo sapiens		*Arabidopsis thaliana*		**OrthoMCL Group**
**EnsEMBL Gene**	**HGNC Symbol**	**TAIR Gene**	**Gene Name**	
ENSG00000065413	ANKRD44	**AT2G03430**	**AT2G03430**	OG5_126538
ENSG00000139645	ANKRD52	AT5G02620	ANK1	
ENSG00000206560	ANKRD28			
ENSG00000164508	HIST1H2AA	AT1G08880	HTA5	OG5_126570
ENSG00000180573	HIST1H2AC	AT1G51060	HTA10	
ENSG00000181218	HIST3H2A	AT1G54690	HTA3	
ENSG00000184260	HIST2H2AC	AT3G20670	HTA13	
ENSG00000184270	HIST2H2AB	AT4G27230	HTA2	
**ENSG00000188486**	**H2AFX**	AT5G02560	HTA12	
ENSG00000196747	HIST1H2AI	AT5G54640	RAT5	
ENSG00000196787	HIST1H2AG	AT5G59870	HTA6	
ENSG00000196866	HIST1H2AD			
ENSG00000203812	HIST2H2AA3			
ENSG00000274997	HIST1H2AH			
ENSG00000276368	HIST1H2AJ			
ENSG00000277075	HIST1H2AE			
ENSG00000278677	HIST1H2AM			
**ENSG00000197061**	**HIST1H4C**	AT1G07660	AT1G07660	OG5_126573
**ENSG00000197238**	**HIST1H4J**	AT1G07820	AT1G07820	
**ENSG00000197837**	**HIST4H4**	AT2G28740	HIS4	
**ENSG00000278705**	**HIST1H4B**	AT3G45930	AT3G45930	
		AT3G46320	AT3G46320	
		AT3G53730	AT3G53730	
		AT5G59690	AT5G59690	
		AT5G59970	AT5G59970	
ENSG00000004700	RECQL	**AT1G10930**	**RECQL4A**	OG5_126644
ENSG00000108469	RECQL5	AT1G31360	MED34	
**ENSG00000165392**	**WRN**	AT1G60930	RECQL4B	
**ENSG00000197299**	**BLM**	AT3G05740	RECQL1	
		AT4G35740	RECQL3	
**ENSG00000150991**	**UBC**	AT1G65350	UBQ13	OG5_126703
**ENSG00000170315**	**UBB**	AT2G35635	RUB2	
		AT3G09790	UBQ8	
		AT4G02890	UBQ14	
		AT4G05050	UBQ11	
		AT4G05320	UBQ10	
		AT5G03240	UBQ3	
		AT5G20620	UBQ4	

Table S9. *Cont.*

**ENSG00000123374**<br>ENSG00000164885	**CDK2**<br>CDK5	AT3G48750	CDKA-1	OG5_126712
ENSG00000103510<br>ENSG00000136504<br>**ENSG00000172977**	KAT8<br>KAT7<br>**KAT5**	AT5G09740<br>AT5G64610	HAM2<br>HAM1	OG5_126735
**ENSG00000177302**	**TOP3A**	AT4G31210<br>AT5G63920	AT4G31210<br>TOP3A	OG5_126736
**ENSG00000051180**<br>**ENSG00000100206**	**RAD51**<br>**DMC1**	AT3G22880<br>**AT5G20850**	DMC1<br>**RAD51**	OG5_126834
**ENSG00000116030**<br>ENSG00000184900<br>**ENSG00000188612**	**SUMO1**<br>SUMO3<br>**SUMO2**	AT4G26840<br>AT5G55160<br>AT5G55856	SUMO1<br>SUMO2<br>SUMO8	OG5_127053
ENSG00000021574<br>**ENSG00000132436**	SPAST<br>**FIGNL1**	AT2G45500<br>**AT3G27120**	AT2G45500<br>**AT3G27120**	OG5_127068
**ENSG00000085999**<br>**ENSG00000197275**	**RAD54L**<br>**RAD54B**	**AT3G19210**	**CHR25**	OG5_127098
ENSG00000071794<br>ENSG00000116830	HLTF<br>TTF2	AT1G02670<br>AT5G05130<br>**AT5G22750**<br>AT5G43530	AT1G02670<br>AT5G05130<br>**RAD5**<br>AT5G43530	OG5_127144
**ENSG00000198793**	**MTOR**	**AT1G50030**	**TOR**	OG5_127212
**ENSG00000143947**	**RPS27A**	AT1G23410<br>AT2G47110<br>AT3G62250	RPS27AA<br>RPS27AB<br>RPS27AC	OG5_127221
ENSG00000039123	SKIV2L2	**AT1G59760**<br>AT1G70070<br>AT2G06990	**AT1G59760**<br>ISE2<br>HEN2	OG5_127259
**ENSG00000136492**<br>**ENSG00000258366**	**BRIP1**<br>**RTEL1**	AT1G20720<br>AT1G20750<br>AT1G79950	AT1G20720<br>AT1G20750<br>AT1G79950	OG5_127294
ENSG00000163170<br>ENSG00000169627<br>**ENSG00000183336**	BOLA3<br>BOLA2B<br>**BOLA2**	AT5G09830	BOLA2	OG5_127446
ENSG00000105011<br>ENSG00000111875	ASF1B<br>ASF1A	**AT1G66740**<br>**AT5G38110**	**ASF1A**<br>**ASF1B**	OG5_127499
**ENSG00000174371**	**EXO1**	AT1G18090<br>AT1G29630	AT1G18090<br>EXO1	OG5_127511
**ENSG00000163312**	**HELQ**	AT4G32700	TEB	OG5_127591
**ENSG00000149923**	**PPP4C**	AT4G26720<br>AT5G55260	PPX1<br>PPX2	OG5_127639
**ENSG00000177889**	**UBE2N**	AT1G16890<br>AT1G78870	UBC36<br>UBC35	OG5_127739
ENSG00000163029	SMC6	**AT5G07660**<br>**AT5G61460**	**SMC6A**<br>**SMC6B**	OG5_127751
**ENSG00000115233**	**PSMD14**	AT5G23540	RPN11	OG5_127757
**ENSG00000175595**	**ERCC4**	**AT5G41150**	**UVH1**	OG5_127977
**ENSG00000221983**	**UBA52**	AT2G36170<br>AT3G52590	RPL40B<br>RPL40B	OG5_128257
**ENSG00000128731**	**HERC2**	AT5G63860	UVR8	OG5_128314
**ENSG00000175054**	**ATR**	**AT5G40820**	**ATR**	OG5_128386

Table S9. *Cont.*

ENSG00000159259	CHAF1B	**AT5G64630**	**FAS2**	OG5_128392
**ENSG00000131153**	**GINS2**	**AT3G12530**	**GINS2**	OG5_128484
**ENSG00000198887**	**SMC5**	**AT5G15920**	**SMC5**	OG5_128615
**ENSG00000012061**	**ERCC1**	**AT3G05210**	**ERCC1**	OG5_128697
**ENSG00000132207**<br>**ENSG00000181625**	**SLX1A**<br>**SLX1B**	AT2G30350	AT2G30350	OG5_128732
ENSG00000014138	POLA2	**AT1G67630**	**POLA2**	OG5_128793
**ENSG00000147536**	**GINS4**	**AT5G49010**	**SLD5**	OG5_128888
**ENSG00000185787**	**MORF4L1**	AT4G37280	MRG1	OG5_128923
**ENSG00000163781**	**TOPBP1**	AT1G77320	MEI1	OG5_128953
**ENSG00000149311**	**ATM**	AT3G48190	ATM	OG5_128955
**ENSG00000125885**	**MCM8**	**AT3G09660**	**MCM8**	OG5_129017
ENSG00000141446<br>ENSG00000171320	ESCO1<br>ESCO2	**AT4G31400**	**CTF7**	OG5_129141
**ENSG00000172732**	**MUS81**	**AT4G30870**<br>AT5G39770	**MUS81**<br>AT5G39770	OG5_129162
**ENSG00000093009**	**CDC45**	**AT3G25100**	**CDC45**	OG5_129331
**ENSG00000152942**	**RAD17**	AT5G66130	RAD17	OG5_129387
**ENSG00000169189**	**NSMCE1**	**AT5G21140**	**emb1379**	OG5_129651
**ENSG00000114742**	**WDR48**	AT3G05090	LRS1	OG5_129752
ENSG00000167670	CHAF1A	**AT1G65470**	**FAS1**	OG5_129755
**ENSG00000151164**<br>**ENSG00000172613**	**RAD9B**<br>**RAD9A**	AT3G05480	RAD9	OG5_129960
**ENSG00000111877**	**MCM9**	AT2G14050	MCM9	OG5_130040
**ENSG00000127922**	**SHFM1**	AT1G64750<br>**AT5G45010**	DSS1(I)<br>**ATDSS1(V)**	OG5_130158
**ENSG00000075131**	**TIPIN**	AT3G02820	AT3G02820	OG5_130239
**ENSG00000128908**	**INO80**	AT3G57300	INO80	OG5_130269
**ENSG00000198690**	**FAN1**	AT1G48360	AT1G48360	OG5_130379
**ENSG00000113456**	**RAD1**	AT4G17760	AT4G17760	OG5_130438
**ENSG00000004487**	**KDM1A**	AT4G16310	LDL3	OG5_130448
**ENSG00000077514**	**POLD3**	**AT1G78650**	**POLD3**	OG5_130668
**ENSG00000139579**<br>**ENSG00000173559**	**NABP2**<br>**NABP1**	AT5G63690	AT5G63690	OG5_130959
**ENSG00000126215**	**XRCC3**	**AT5G57450**	**XRCC3**	OG5_131019
**ENSG00000108384**	**RAD51C**	AT2G45280	RAD51C	OG5_131246
**ENSG00000156831**	**NSMCE2**	**AT3G15150**	**MMS21**	OG5_131427
**ENSG00000111602**	**TIMELESS**	AT5G52910	ATIM	OG5_131860
**ENSG00000139618**	**BRCA2**	**AT4G00020**<br>**AT5G01630**	**BRCA2A**<br>**BRCA2B**	OG5_131863
**ENSG00000163605**	**PPP4R2**	AT5G17070	AT5G17070	OG5_132213
**ENSG00000100811**<br>ENSG00000230797	**YY1**<br>YY2	AT4G06634	AT4G06634	OG5_132824
**ENSG00000185379**	**RAD51D**	AT1G07745	RAD51D	OG5_132909
ENSG00000152253	SPC25	**AT3G48210**	**AT3G48210**	OG5_132949
**ENSG00000104320**	**NBN**	**AT3G02680**	**NBS1**	OG5_133106
**ENSG00000196584**	**XRCC2**	**AT5G64520**	**XRCC2**	OG5_133234
**ENSG00000214941**	**ZSWIM7**	**AT4G33925**	**AT4G33925**	OG5_133488
**ENSG00000178295**	**GEN1**	AT1G01880	GEN1	OG5_134343
**ENSG00000063978**	**RNF4**	AT3G07200	AT3G07200	OG5_134631
**ENSG00000182185**	**RAD51B**	**AT2G28560**	**RAD51B**	OG5_135350
**ENSG00000158019**	**BRE**	AT5G42470	AT5G42470	OG5_135444
**ENSG00000175643**	**RMI2**	AT1G08390	AT1G08390	OG5_139541

Table S10. NHEJ genes in human and *Arabidopsis thaliana,* grouped according to orthology.

Homo sapiens		*Arabidopsis thaliana*		**OrthoMCL Group**
**EnsEMBL Gene**	**HGNC Symbol**	**TAIR Gene**	**Gene Name**	
**ENSG00000129484**<br>**ENSG00000143799**	**PARP2**<br>**PARP1**	AT2G31320<br>**AT4G02390**	PARP1<br>**PARP2**	OG5_128423
**ENSG00000166896**	**XRCC6BP1**	AT3G03420	AT3G03420	OG5_128965
**ENSG00000196419**	**XRCC6**	**AT1G16970**	**KU70**	OG5_129086
**ENSG00000079246**	**XRCC5**	**AT1G48050**	**KU80**	OG5_129372
**ENSG00000124795**	**DEK**	AT3G48710<br>AT4G26630<br>AT5G55660<br>AT5G63550	AT3G48710<br>AT4G26630<br>AT5G55660<br>AT5G63550	OG5_130851
**ENSG00000198382**	**UVRAG**	AT2G32760	AT2G32760	OG5_131718
**ENSG00000073050**	**XRCC1**	**AT1G80420**	**XRCC1**	OG5_132817
**ENSG00000152422**	**XRCC4**	AT3G23100	XRCC4	OG5_135131
